# The role of the gut microbiome in the development of hepatobiliary cancers

**DOI:** 10.1097/HEP.0000000000000406

**Published:** 2023-04-15

**Authors:** Neil Daniel, Flavia Genua, Mazda Jenab, Ana-Lucia Mayén, Anastasia Chrysovalantou Chatziioannou, Pekka Keski-Rahkonen, David J. Hughes

**Affiliations:** 1 Cancer Biology and Therapeutics Laboratory, Conway Institute, School of Biomedical and Biomolecular Sciences, University College Dublin, Dublin, Ireland; 2 Nutrition and Metabolism Branch, International Agency for Research on Cancer, Lyon, France

## Abstract

Hepatobiliary cancers, including hepatocellular carcinoma and cancers of the biliary tract, share high mortality and rising incidence rates. They may also share several risk factors related to unhealthy western-type dietary and lifestyle patterns as well as increasing body weights and rates of obesity. Recent data also suggest a role for the gut microbiome in the development of hepatobiliary cancer and other liver pathologies. The gut microbiome and the liver interact bidirectionally through the “gut-liver axis,” which describes the interactive relationship between the gut, its microbiota, and the liver. Here, we review the gut-liver interactions within the context of hepatobiliary carcinogenesis by outlining the experimental and observational evidence for the roles of gut microbiome dysbiosis, reduced gut barrier function, and exposure to inflammatory compounds as well as metabolic dysfunction as contributors to hepatobiliary cancer development. We also outline the latest findings regarding the impact of dietary and lifestyle factors on liver pathologies as mediated by the gut microbiome. Finally, we highlight some emerging gut microbiome editing techniques currently being investigated in the context of hepatobiliary diseases. Although much work remains to be done in determining the relationships between the gut microbiome and hepatobiliary cancers, emerging mechanistic insights are informing treatments, such as potential microbiota manipulation strategies and guiding public health advice on dietary/lifestyle patterns for the prevention of these lethal tumors.

## Graphical abstract

**Figure:**
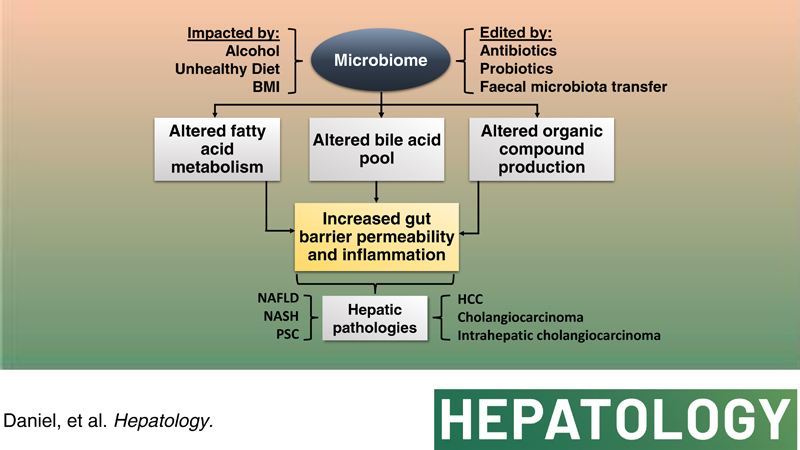

## INTRODUCTION

Primary liver cancers, of which 90% present as HCC, rank sixth in global cancer incidence and have very high morbidity and mortality.^1^ Tumors of the biliary tract (ie, intrahepatic, extrahepatic, and gall bladder) are in close anatomic proximity to the liver and, together with HCC, are considered hepatobiliary cancers (HBCs).^2^ Globally, HCC is the third leading cause of cancer death with upwards of 800,000 deaths reported in 2020.^3^ These tumors are associated with poor clinical outcomes due to high levels of invasiveness, propensity for distant metastasis, and resistance to existing treatments.^4^ Cholangiocarcinoma (CCA) is the second most common primary hepatic malignancy, where most patients present with unresectable disease and poor prognosis.^5^ Existing evidence indicates that HBCs are initiated and progress through the accumulation of genetic and epigenetic lesions, most commonly in the *TP53*, *CTNNB1*, *AXIN1*, *ARID1A,* and *WWP1* genes.^6^ These lesions are influenced by obesity, unhealthy dietary and lifestyle patterns, HCV and HBV status, alcohol consumption, and ingestion of fungal aflatoxin B1.^4^ These exposures contribute to a range of liver pathologies such as cirrhosis, alcohol-associated liver disease, alcohol-associated steatohepatitis, nonalcoholic fatty liver disease (NAFLD), nonalcoholic steatohepatitis (NASH), and HBC.

Recent convincing evidence suggests that the gut microbiome plays an important role in hepatocarcinogenesis.^7^ A healthy microbiome (in eubiosis) is characterized by the prevalence of *Bacteroidetes* and *Firmicutes*, with smaller abundances of *Proteobacteria*, *Actinobacteria,* and *Verrucomicrobia.* 8 The shift of bacterial composition from eubiosis to a state of “dysbiosis” is characterized by a disruption to the gut microbiota homeostasis and is caused by an imbalance in the microflora, an alteration of their activities or function, or a shift in their local distribution.^9^ Intestinal dysbiosis has been linked with a myriad of conditions ranging from obesity to atherosclerosis and tumor development at several anatomic sites,^10^–^12^ including the liver and biliary tract.

The gut and liver engage in reciprocal communication through 3 avenues: the biliary tract, the portal vein, and the systemic circulation.^13^ In this review, we outline the experimental evidence for the role of dysbiosis and exposure to endogenous toxins and metabolites generated or modified by bacteria as contributors to the development of HBC. We also summarize the latest findings regarding the impact of lifestyle factors on liver health as mediated by the gut microbiome and emerging strategies for gut biome editing.

### Dysbiosis-driven inflammation

Bacterial translocation is a phenomenon whereby low levels of viable microbes and other inert particles and macromolecules such as lipopolysaccharide (LPS), flagellin, colibactin, and mRNA escape the intestinal lumen.^14^ Normally, these microbe-associated molecular patterns are identified through interaction with toll-like receptors (TLRs) and nucleotide-binding oligomerization domain-like receptors and then transported to the mesenteric lymph nodes by dendritic cells for priming of the adaptive immune system.^15^ Even in healthy individuals, trace amounts of these bacterial components evade capture by host immune cells and reach the liver through the portal vein. Kupffer cells, the largest resident macrophage population in the liver, phagocytose microbes derived from the bloodstream and may act as a firewall between host and commensal microorganisms, as demonstrated in mouse models.^16^

However, after an increase in intestinal permeability, mesenteric lymph nodes may become unable to clear microbe-associated molecular patterns, metabolites, and whole bacteria, resulting in these elements reaching the liver through the portal circulation. This sustained insult to the liver triggers an inflammatory response. Kupffer cells sit at the heart of this response; following stimulation of TLRs by LPS, the proinflammatory NF-κB pathway is activated and TNFα, IL1β, IL6, IL12, IL18, and several chemokines including CXCL1, CXCL2, CCL2, CCL5, CCL3, and CCL4, are produced, along with the resultant production of reactive oxygen species.^17^–^19^ This local inflammation leads to the recruitment of neutrophils, CD4+ T cells, monocytes, and other leukocytes, which further contribute to inflammation ultimately leading to necrosis, apoptosis, and over time, neoplasia.^20^,^21^ The action of Kupffer cells and the liver’s ability to deal with translocated bacteria and leaked toxins is an element of liver functionality. In those where liver function is compromised, there is increased leakage of bacterial products into the systemic circulation, potentially affecting not only other organs but also prompting systemic inflammation and promoting metabolic dysfunction, which could then further negatively impact both the liver and biliary tract.^22^

In mouse models of HCC, it has been reported that dysbiosis precedes liver inflammation. This was followed by further compositional and functional shifts in the microbiome resulting in gut barrier dysfunction along with the development of cirrhosis and HCC. Increases in serum LPS and serum IFNγ, TNFα, IL6, and IL17 were also observed, supporting the hypothesis that gut dysbiosis-dependent events and related inflammatory responses act synergistically to promote HCC development.^23^ The microbiome profiles that may contribute to these mechanisms vary across studies and are discussed in the following (findings are summarized in Table 1).

**TABLE 1:** Changes in bacterial gut populations in liver cancer

Condition	Microbes	Method	Reference
HCC vs healthy controls	*Ruminococcus* ↑ *Oscillibacter* ↑ *Faecalibacterium* ↑ *Clostridium IV* ↑ *Coprococcus* ↑ *Klebsiella* ↓ *Haemophilus* ↓	Fecal 16S rRNA sequencing (HCC n = 75, healthy controls n = 105)	24
Cirrhosis + HCC vs cirrhosis	*Fusobacteria* ↑ *Odoribacter* ↑ *Prevotella* ↓ *Dorea* ↓	Fecal 16S rRNA sequencing (Cirrhosis + HCC n = 25, Cirrhosis = 25)	25
	*Bacteroides* ↑ *Bifidobacterium* ↓ *Akkermansi*a ↓	Metagenomic analysis of stool DNA (Cirrhosis + HCC n = 21, Cirrhosis n = 20)	26
	*Clostridium sensu stricto* ↓ *Anaerotruncus* ↓ *Raoultella* ↑ *Haemophilus* ↑	Prospective 16s rRNA sequencing (Cirrhosis leading to HCC n=33, Cirrhosis=33)	27
NAFLD-HCC vs non-HCC	*Bacteroides caecimuris* ↑ *Veillonella parvula* ↑	Fecal 16S rRNA sequencing (NAFLD-HCC n = 32, non-HCC controls n = 58)	28
HCC vs Hep B related HCC	*Escherichia-Shigella* ↑ *Enterococcus* ↑ *Faecalibacterium* ↓ *Ruminococcus* ↓ *Ruminoclostridium* ↓	Fecal 16S rRNA sequencing (Hep B related HCC n = 35, HCC n = 22)	29
ICC vs healthy controls	*Lactobacillus* ↑ *Actinomyces* ↑ *Peptostreptococcaceae* ↑ *Alloscardovi* ↑	Fecal 16S rRNA sequencing (ICC n = 28, healthy controls n = 12)	30

Abbreviation: ICC, intrahepatic cholangiocarcinoma.

## GUT MICROBIOME AND HEPATOBILIARY CARCINOGENESIS

The effect of the gut microbiome on hepatobiliary conditions has become an emerging area of interest. Recent decades have seen an increase in the prevalence of NAFLD, which refers to a spectrum of liver conditions ranging from simple steatosis to a highly inflammatory condition known as NASH. NASH is characterized by hepatic steatosis along with liver cell ballooning, steatohepatitis, and varying degrees of fibrosis, which may lead to cirrhosis.^31^ Each form of liver disease has been associated with a unique microbiome, for example, a metagenomic study conducted using stool sample DNA observed that *Eubacterium rectale* and *Bacteroides vulgatus* were the most abundant species in mild/moderate NAFLD, while *Bacteroides vulgatus* and *Escherichia coli* were the most abundant in patients with NAFLD and advanced fibrosis. In addition, *Ruminococcus obeum* and *Eubacterium rectale* were significantly lower in advanced fibrotic NAFLD than mild/moderate NAFLD.^32^

Using metagenomic analysis of stool samples from 27 patients with NAFLD-cirrhosis and 54 non-NAFLD controls, Oh et al identified a 19-species microbiome signature for NAFLD-cirrhosis. The signature, when combined with age and serum albumin levels, yielded a receiver operating characteristic AUC of 0.91 and included increases in the levels of *Veillonella parvula, Veillonella atypica, Ruminococcus gnavus, Clostridium bolteae,* and *Acidaminococcus sp*. D21 and decreases in the abundances of *Eubacterium eligens, Eubacterium rectale,* and *Faecalibacterium prausnitzii*. Interestingly, this signature was capable of identifying patients with cirrhosis in 2 independent Chinese and Italian cohorts, with respective AUCs of 0.86 and 0.89.^33^

In a prospective study, Leung et al performed metagenomic and metabolomic analyses of stool and serum samples in a nested case-control study of 90 participants with NAFLD (at follow-up after 4.6 y) matched to 90 disease-free controls. The authors then used machine learning to build a prospective model composed of 2 genera (*Methanobrevibacter* and *Slackia*), 3 pathways, 9 metabolites, and 4 anthropometric and clinical characteristics that enabled stratification of prediagnostic NAFLD cases and controls with an AUC of 0.80.^34^

A 16s rRNA (16s) pyrosequencing study showed that patients with NASH had a differential abundance of *Faecalibacterium* and *Anaerosporobacter* (lower) and *Parabacteroides* and *Allisonella* (higher).^35^ Another 16s study, in addition, revealed that patients with NASH displaying stage 2 fibrosis or higher also had increases in bacterial genera *Bacteroides* and reduction in *Prevotella.* 36 Insight into how the microbiome may modulate liver carcinogenesis from liver disease was provided in a further metagenomic analysis of stool samples,^28^ which reported that *Bacteroides caecimuris* and *Veillonella parvula* were both significantly enriched in NAFLD-HCC compared with NAFLD-cirrhosis and non-NAFLD controls as well as being capable of discriminating between the former and latter 2. Bacterial extracts from the NAFLD-HCC microbiota, but not from the control groups were then seen to promote a T cell immunosuppressive phenotype, characterized by expansion of regulatory T cells and attenuation of CD8+ T cells in ex vivo models.^28^

A parallel path to carcinogenesis, alcohol-associated liver disease is also characterized by steatosis, eventually leading to alcohol-associated steatohepatitis.^37^ The gut bacterial populations of patients with alcoholism display reduced proportions of *Bacteroidaceae* compared with controls and patients with alcohol-associated cirrhosis have increases in the *Prevotellaceae* family compared with patients with hepatitis *B*-related cirrhosis.^38^,^39^ 16s sequencing also revealed that patients with the alcohol-associated liver disease exhibit duodenal dysbiosis with increases in *Streptococcus, Shuttleworthia,* and *Rothia* compared with matched controls.^40^ The mycobiome, which refers to the diverse array of fungal species found in tissue, has also been seen to be altered in fecal samples from patients with alcohol-dependence, alcoholic hepatitis, or alcohol-associated cirrhosis compared with controls. Besides displaying diminished fungal diversity and *Candida* overgrowth, alcohol-associated disease mouse models exhibited increased systemic immune responses to mycobiota such as increased IL1β expression by Kupffer cells.^41^

A 16s rRNA sequencing study conducted by Ponziani et al showed that the abundances of *Bacteroides* were increased and *Bifidobacterium* and *Akkermansia* reduced in 21 patients with cirrhotic HCC relative to 20 patients with cirrhosis but without HCC. The authors also found elevated levels of fecal calprotectin and plasma concentrations of IL8, IL13, CCL3, CCL4, and CCL5 in the patients with HCC. *Akkermansia* and *Bifidobacterium* abundances were inversely correlated with calprotectin levels while the increase in *Bacteroides* abundance was associated with increased levels of IL8 and IL13 and activated circulating monocytes.^26^

A similar study also compared the gut microbiomes of 25 patients with cirrhotic HCC, 25 patients with cirrhosis, and 25 controls. A 3-fold enrichment of Erysipelotrichaceae and a 5-fold decrease in the Leuconostocaceae families were found in the HCC group relative to patients with cirrhosis but without HCC. They also found a 5-fold decrease in *Fusobacteria* coupled with an increase in the *Bacteroides*/*Prevotella* ratio in patients with cirrhotic HCC versus patients with cirrhosis only. The authors also noted a trend of increasing IL6 and TNFα concentrations from controls to cirrhosis to HCC but attribute the lack of significance to the small sample size.^25^

A prospective study of patients with cirrhosis compared stool samples from 33 who subsequently developed HCC to an equal number who remained cancer free. Higher relative abundances of *Clostridium sensu stricto* and *Anaerotruncus* (ascertained by 16s sequencing) were significantly associated with protection from HCC, whereas *Raoultella* and *Haemophilus* were positively associated with HCC.^27^

Using a metagenomic approach in stool samples, a recent study analyzed a cohort of 35 patients with HBV-related HCC and 22 patients with HCC without prior HAV/HBV infection, plus 33 controls. It was found that the feces of the patients with HCC but without HAV/HBV infection harbored more bacteria known to have a proinflammatory role such as *Escherichia-Shigella* and *Enterococcus* while levels of anti-inflammatory short-chain fatty acid (SCFA) producing bacteria, such as *Faecalibacterium*, *Ruminococcus,* and *Ruminoclostridium,* were reduced.^29^

Recently, fecal 16S rRNA sequencing and random forest algorithm analysis performed in a cohort of 40 healthy volunteers, 143 patients with HCC, and 46 patients with CCA identified an 8 genera signature consisting of *Faecalibacterium, Klebsiella, Ruminococcus gnavus group, Lactobacillus, Dorea, Veillonella, Burkholderia Caballeronia Paraburkholderia,* and *Citrobacter* that was able to discriminate between healthy controls, patients with HCC and patients with CCA with AUCs of 0.989, 0.967, and 0.920, respectively.^42^

Although there is considerable available literature illustrating the differences between the gut populations of controls, patients with cirrhosis, and patients with HCC, the bacterial profiles are highly variable between studies. Larger studies are needed to determine whether a consensus diagnostic/prognostic microbiome for HBCs can be defined. One such study analyzed 419 fecal samples to create a panel of 30 16S rRNAs capable of discriminating between 75 early HCC and 105 non-HCC samples in a validation cohort, generating an AUC of 80.64%. Butyrate (an SCFA that is thought to have anticancer properties) producing bacteria such *as Ruminococcus, Oscillibacter, Faecalibacterium, Clostridium IV,* and *Coprococcus* were reduced whereas LPS-producing genera such as, *Klebsiella* and *Haemophilus,* were increased.^43^,^44^

Similarly, a nested case-control study conducted in a cohort of Finnish male smokers of 224 liver cancer cases and matched controls found that the cases had increased immune responses to circulating bacterial translocation markers LPS, flagellin, soluble CD14, and LPS-binding protein.^45^ Furthermore, a second case-control study nested within the large European Prospective Investigation into Cancer and Nutrition (EPIC) cohort also found a combination of antibody responses to LPS and flagellin to be associated with an increased risk of HCC, with an odds ratio (OR) point estimate of 11.76 when comparing the highest and lowest quartiles, though with wide confidence intervals and a higher disease risk observed for men than women. However, while the authors discussed the plausibility of a potential sex-specific effect, they noted that this stratified finding lacked statistical significance due to the small number of female HCC cases within the study.^46^

These larger studies corroborate the results of smaller studies indicating that disruptions to gut populations cause translocation and an increase in proinflammatory LPS leading to the activation of TLR4 and NF-κB and the release of proinflammatory cytokines TNFα, IL6, and IL1. In tandem, bacterial species that produce butyrate are diminished and gut integrity is compromised.^47^,^48^

Petrick et al, assessed the associations between HCC and circulating markers of gut barrier integrity: Ig IgA, IgG, and IgM against LPS and flagellin, soluble CD14, and LPS-binding protein in serum taken prediagnostically from 281 cases and 257 controls across the Taiwanese REVEAL‐HBV/HCV cohorts. A doubling of the serum concentrations of IgA against flagellin or LPS-binding protein was associated with 76% and 93% increased risks of HBV-related HCC, respectively. No association was reported between HCV-related HCC and the assessed markers.^24^

These results, while encouraging and accompanied by a plausible mechanism of action, need to be reproduced in alternate populations and in further prospective settings such as the aforementioned Finnish, European, and Taiwanese studies, as well as mechanistic studies to help address possible reverse-causality from the case-control studies.

The bile duct delivers bile fluids directly from the liver and gall bladder to the intestine. Malignant transformation of biliary tree epithelial cells known as cholangiocytes, results in CCA.^49^ Although a distinct, tissue-specific biome predominantly composed of bacterial families *Dietziaceae, Pseudomonadaceae,* and *Oxalobacteraceae* has been noted in cases of CCA, the potential role of the gut microbiome has been little studied.^50^ A 2019 study of 28 cases of HCC, 12 controls, and 28 cases of intrahepatic cholangiocarcinoma found that at the genus level, the gut microbiota found in the stool of patients with intrahepatic cholangiocarcinoma contained higher levels of *Lactobacillus, Actinomyces, Peptostreptococcaceae,* and *Alloscardovia* relative to the other patients.^30^

Colitis and primary sclerosing cholangitis (PSC), a chronic inflammatory liver disease that leads to scarring of the bile ducts and biliary tree of the liver, are considered important risk factors for CCA development.^51^ A recent study reported a reduction in gut barrier function in mice with PSC and colitis as assessed by a fluorescein isothiocyanate-dextran permeability assay. This allowed the translocation of bacteria and LPS to the liver, inducing myeloid-derived suppressor cells, resulting in an immunosuppressive environment promoting carcinogenesis.^52^

### Microbial metabolites and hepatobiliary carcinogenesis

#### Bile acids

**FIGURE 1:**
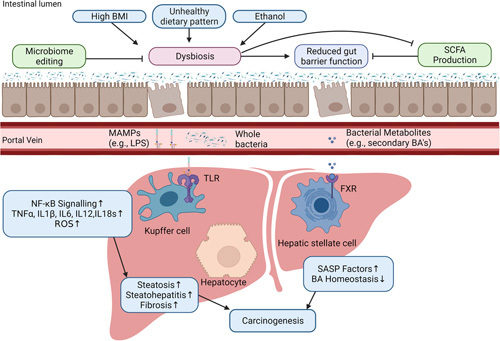
The gut-liver axis. Unhealthy dietary/lifestyle exposures may lead to gut microbiome disturbances (and potentially pathogenic dysbiosis) with resultant alterations in SCFA concentrations and reduced gut barrier integrity, allowing leakage of bacterial products, such as LPS, and translocation of bacteria toward the liver through the portal vein and systemic circulation, where they are bound by TLRs causing an increase in NF-kB–mediated inflammatory cytokine and ROS production. Dysbiosis also causes an increase in secondary BAs, the majority of which return to the liver through the portal vein and may disrupt BA homeostasis by binding FXR. These mechanisms are thought to increase steatosis and steatohepatitis, and ultimately impact carcinogenesis. Abbreviations: BA, bile acid; BMI, body mass index; FXR, Farnesoid *X* receptor; LPS, lipopolysaccharide; MAMP, microbe-associated molecular pattern; ROS, reactive oxygen species; SASP, secretory associated senescent phenotype; SCFA, short-chain fatty acid; TLR, toll-like receptor.

Bile acids (BAs) are cholesterol derivatives produced in the liver and stored in the gall bladder. These molecules not only have functions in digestion but also are regulators of inflammation and microbial populations.^53^ BAs were first implicated in carcinogenesis in 1940 and have subsequently been shown to be involved in multiple gastrointestinal tumors.^54^ Primary BAs are synthesized by 4 pathways: classic, alternative, Yamasaki, and 25-hydroxylation pathways, which comprise 17 enzymes.^55^ The primary products are cholic acid and chenodeoxycholic acid, which are then conjugated to glycine or taurine. After secretion, the majority (95%) of BAs are transported back to the liver through the portal vein.^56^,^57^ The remaining 5% are metabolized by the gut microbiota: deconjugated by bacterial bile salt hydrolase, and dehydrogenated, and dehydroxylated to create secondary BAs, such as deoxycholic acid (DCA), which are then passively absorbed into the portal circulation and back to the liver, as illustrated in Figure 1.^56^,^57^ BAs act as signaling molecules by interacting with the Farnesoid *X* receptor and *G*-protein coupled BA receptor 1.^58^,^59^ BA homeostasis is disturbed by dysbiosis, altering the BA pool, which in turn may further increase dysbiosis.^60^,^61^ How dysbiosis-driven alterations of BA profiles affect hepatobiliary health is a subject of current investigation. BAs affect cellular phenotype and both immune signaling molecules and effectors. Alterations to the BA pool and Farnesoid *X* receptor expression levels have been observed in case-control studies of HCC, gall bladder cancer, and CCA.^62^–^66^

In vitro, DCA was seen to induce a secretory associated senescent phenotype (SASP) in mouse hepatic stellate cells.^67^ Secreted SASP factors can stimulate tumor cell proliferation, migration, and invasion.^68^ When treated with the conditioned media of mouse hepatic stellate cells exposed to DCA, HCC cells underwent epithelial-to-mesenchymal transition with increased migration and invasion. Subsequently, in the HCC cohort of the cancer genome atlas database, the authors found that SASP factor expression in human HCC tissue was correlated with invasion markers and poor survival.^67^

Similarly, obese mouse hepatic stellate cells treated with DCA and bacterial cell component lipoteichoic acid showed increased expression of SASP factors and COX2-mediated prostaglandin E2 production, which reduced activity in liver-derived immune cells thereby increasing tumor immune evasion. Furthermore, COX2 overexpression and increased prostaglandin E2 production have been observed in human HCC tissue with NASH implicating similar mechanistic responses in humans.^69^,^70^

After the ablation of BA metabolizing bacteria such as *Clostridium* in mice, more primary BAs were returned to the liver resulting in increased production of CXCL16 by liver endothelial cells. This caused an influx of natural killer T cells, increasing intrahepatic levels of IFNγ with lower levels of HCC and hepatic metastasis observed in treated mice relative to controls.^71^ In addition, blocking DCA production by decreasing enzymatic 7α-dehydroxylation reduces HCC development in obese mice.^72^

Untargeted metabolomics in a 1:1 matched nested prospective case-control study of liver cancer (n = 221 cases) and fatal liver disease (n = 242 cases) within the Alpha-Tocopherol, Beta-Carotene Cancer Prevention Study revealed that higher serum levels of conjugated primary BA glycocholic acid (GCA) and glycochenodeoxycholic acid (GCDCA) were associated with an elevated risk of liver cancer in Finnish male smokers.^73^ Similar positive associations of serum GCA and GCDCA with HCC risk were observed in another prospective study that included 129 HCC case-control pairs nested in the EPIC cohort.^74^

In concordance with the above studies, Petrick et al, quantified 15 circulating BAs in serum taken prediagnostically from 281 HCC cases and 257 matched controls across the REVEAL‐HBV/HCV cohorts and found that higher levels of glycine and taurine-conjugated primary BAs taurocholic acid, GCA and GCDCA were associated with a 2–8‐fold increase in the risk of HBV and HCV associated HCC. Higher circulating levels of the secondary BA DCA were associated with a decreased HCC risk.^75^

Similar findings were recently reported from the Singapore Chinese Health Study after the quantification of 35 BAs in the serum of 100 HCC cases taken ~4 years before diagnosis and 100 matched controls. Conjugated primary BAs taurochenodeoxycholic acid, taurocholic acid, GCA, and GCDCA were significantly elevated in the HCC cohort, while the ratios of secondary BAs over primary BAs were significantly lower in HCC cases than in controls.^76^

Finally, a prospective study, including 233 HCC case-control pairs nested within the EPIC cohort, performed targeted metabolomics to quantify 17 BAs in prediagnostic plasma. Circulating major BA levels, particularly GCA and taurine-conjugated BAs, were strongly associated with HCC development.^77^

Although the evidence is mounting that BAs affect carcinogenesis in hepatocytes, cholangiocytes, and intestinal epithelial cells, their effects on other hepatobiliary tissues have been little studied. However, a recent study involving xenograft mouse models showed that DCA was downregulated in gall bladder cancer, while DCA treatment inhibited tumor growth. Furthermore, higher DCA concentrations were significantly correlated with favorable clinical outcomes in patients with gall bladder cancer.^78^ These studies highlight the necessity for further studies on the effect of the microbiome on the BA pool, as altered concentrations of BAs have been associated with both increased and decreased risk of carcinogenesis.

#### Amino acid derivatives

Although BAs play a central role in hepatobiliary carcinogenesis, other metabolites are also involved in the promotion of or protection against pathologic processes within the liver (summarized in Table 2). Amino acid derivatives such as phenylacetic acid, imidazole propionate, and 3-(4-hydroxyphenyl)lactate have been identified as potential inducers of steatosis and hepatic inflammation.^83^ Amino acids such as tryptophan, phenylalanine, and tyrosine have also recently been suggested to be involved in the development of NAFLD.^84^ For branched-chain amino acids, they inhibit the incidence of HCC in patients with cirrhosis and obesity.^85^ Higher plasma and urine concentrations are found in patients with NAFLD while plasma phenylacetic acid can significantly increase branched-chain amino acid hepatic utilization and facilitate hepatic lipid accumulation.^86^,^87^

**TABLE 2:** Changes in microbial-derived metabolites in liver cancer and liver disease

Classes of metabolites and metabolites	Condition	Elevated or Decreased levels in cases	Sample type	Reference
Bas GCA and GCDCA Conjugated primary BAs GCA, TCA and GCDCA, TCDCA Secondary BAs DCA	Liver cancer (n = 221) vs controls (n = 221) Fatal liver disease (n = 242) vs controls (n = 242) HCC cases (n = 129) vs controls (n = 129) HBV-related HCC cases (n = 185) vs controls (n = 161) Pre-diagnostic HCC (n = 100) vs controls (n = 100) Pre-diagnostic HCC (n = 233) vs controls (n = 233) HCV-related HCC cases (n = 96) vs controls (n = 96) HBV-related HCC cases (n = 185) vs controls (n = 161)	Elevated Elevated Elevated Elevated Elevated Elevated Elevated Decreased	Blood serum Blood serum Blood serum Blood serum Blood serum Blood serum Blood serum Blood serum	73 73 74 75 76 77 75 75
SCFAs and intermediates				
Acetate, butyrate, formate, oxaloacetate, acetylphosphate	NAFLD-HCC (n = 32) vs NAFLD-cirrhosis (n = 28) and non-NAFLD control (n = 30)	Elevated	Feces	28
Butyrate, propionate, malonate Isocitrate	NAFLD-HCC (n = 32) vs NAFLD-cirrhosis (n = 28) and non-NAFLD control (n = 30) NAFLD-HCC (n=32) vs NAFLD-cirrhosis (n=28) and non-NAFLD control (n=30)	Elevated Decreased	Blood serum Feces	28 28
Ethanol (bacteria-produced)	NASH (n = 22) vs obese non-NASH (n = 25) and controls (n = 16)	Elevated	Blood serum	79
TMAO	NAFLD (n = 60) vs controls (n = 35) NAFLD (n = 914) vs controls (n = 714) NASH (n = 109) vs non-NASH (n = 126)	Elevated Elevated Elevated	Blood serum Blood serum Blood serum	80 80 81
Choline	NAFLD (n = 60) vs controls (n = 35) NASH (n = 109) vs non-NASH (n = 126)	Elevated Elevated	Blood serum Blood serum	80 81
TMAVA	Liver steatosis (n = 15) vs healthy 15 Fatty liver disease (n = 273) vs controls (n = 494)	Elevated Elevated	Blood plasma Blood plasma	82 82

Abbreviations: BA, bile acid; DCA, deoxycholic acid; GCA, glycocholic acid; GCDCA, glycochenodeoxycholic acid; SCFA, short-chain fatty acid; TCA, taurocholic acid; TCDCA, taurochenodeoxycholic acid; TMAO, trimethylamine-*N*-oxide; TMAVA, *N,N,N*-trimethyl-5-aminovaleric acid.

In contrast, gut bacteria-derived metabolites of tryptophan; indole-3-propionic acid, indole-3-acetic acid, indole-3-aldehyde, tryptamine, and 3-methylindole have been found to maintain intestinal integrity, reduce bacterial translocation and prevent the leakage of microbiota-derived components from the intestines to the liver, limiting local and distant inflammatory cascades.^88^ Circulating indole-3-propionic acid levels have been observed to be lower in individuals with liver fibrosis compared with those without fibrosis.^89^ Indole-3-acetate also has protective hepatic effects that attenuate inflammatory responses and reduce lipid accumulation in hepatocytes.^90^ Also, *L*-glutamine, the most plentiful amino acid in the body, improves intestinal barrier functioning and decreases the occurrence of infection.^91^

#### Fatty acids

SCFAs, saturated fatty acids with a short carbon chain (1–6 carbon atoms), are a category of metabolites that are mainly produced in the intestine by gut microbes as a result of the fermentation of dietary fibers and starches that are resistant to digestion. A recent study, performing metabolomics to characterize NAFLD-associated cirrhosis, with and without HCC, showed that the levels of acetate, butyrate, and formate were elevated in the feces of patients with NAFLD-HCC compared with NAFLD-cirrhosis and non-NAFLD controls, while the serum levels of butyrate, propionate, and malonate were also elevated in the same group.^28^ SCFAs reduce hepatic cholesterol and fatty acid synthesis but increase hepatic lipid oxidation.^86^ They participate in fatty acid β-oxidation and insulin sensitivity that are both closely linked to NAFLD.^92^ They also have anti-inflammatory effects by reducing migration and proliferation of immune cells, and pro-inflammatory cytokines and up-regulate the anti-inflammatory cytokine prostaglandin E2.^86^ SCFAs protect the liver by maintaining gut barrier homeostasis and reducing pro-inflammatory cytokine secretion.^93^ They are an energy source for gut enterocytes and stimulate the tight junctions of the gastrointestinal barrier thereby enhancing gut barrier functionality.^94^

Omega-3 polyunsaturated fatty acids directly impact the composition and diversity of gut microbiota, for example, by lowering the abundance of pro-inflammatory bacteria (eg, Proteobacteria) and increasing anti-inflammatory bacteria (eg, *Bifidobacterium and Akkermansia muciniphila*).^95^ Omega-3 polyunsaturated fatty acids contribute to the integrity of intestinal mucosa and reduce intestinal permeability and bacterial translocation.^96^,^97^ Also, dietary omega-3 polyunsaturated fatty acid supplementation promotes a decrease in LPS and trimethylamine concentrations in plasma.^91^

#### Organic compounds

Trimethylamine can be oxidized to trimethylamine-*N-*oxide (TMAO) and increase insulin resistance, promote inflammation and oxidative stress.^98^ Higher serum levels of TMAO are correlated with greater severity of NAFLD^80^ and NASH, but only in patients with type 2 diabetes after adjusting for insulin resistance in a cross-sectional study.^81^ Notably, TMAO has been recently reported to increase with body mass index and is positively associated with the visceral adiposity index, as well as the fatty liver index, which could be used to predict the presence of NAFLD.^99^ Also, *N,N,N*-trimethyl-5-aminovaleric acid, a gut microbiota metabolite, exacerbates liver steatosis through the inhibition of carnitine synthesis and a reduction in mitochondrial fatty acid β-oxidation in hepatic tissue.^82^

Ethanol is naturally produced by the gut microbiota and metabolized by the enzymes of the liver. Certain high-alcohol-producing bacteria have been identified and some of them have been studied in the context of gut fermentation syndrome.^91^ Patients with NASH have been found to have increased production of endogenous ethanol compared with controls, due to a higher abundance of alcohol-producing bacteria.^79^ In addition, a recently studied bacteria, namely *Klebsiella pneumoniae,* has been associated with up to 60% of individuals with NAFLD in a Chinese cohort.^100^ However, for patients with NAFLD, the source of endogenous ethanol remains equivocal.^101^ Elevated levels of ethanol are able to downregulate tight junction expression promoting intestinal barrier leakage, up-regulate de novo lipogenesis, decrease fatty acid oxidation, and reduce VLDL exportation from the liver, contributing to a higher NAFLD risk.^86^ Dysbiosis possibly promotes an increase in the production of endogenous ethanol by the gut microbiome, contributing to liver pathogenesis.^86^ However, the role of the gut microbiota in the production of endogenous ethanol and their consequent impact on NAFLD needs to be further investigated.

## LIFESTYLE FACTORS IN HEPATOBILIARY DISEASE

### Diet

Unhealthy dietary patterns are thought to be obesogenic, pro-inflammatory, and promote gut microbiome dysbiosis, which, as outlined, increases intestinal permeability and is associated with hepatobiliary disease.^102^ Diets containing vegetables, fiber, wholegrains, fish, poultry, coffee, monounsaturated fatty acids, and high levels of micronutrients such as vitamin E, vitamin B9, β-carotene, manganese, and potassium have been associated with reduced risk of HCC.^103^ The western diet is high in sugar and saturated fat, both established contributors to gut microbiome dysbiosis.^104^ The interactions between dietary patterns, the gut microbiome, and their downstream effect on hepatobiliary health are less understood.

In mouse models subjected to chronic or sporadic dietary fiber deficiency, the gut microbiota metabolizes host-produced mucus glycoproteins as an alternate nutrient source leading to a degradation of the colonic mucus barrier.^105^ Similarly, mice fed with a western-style diet displayed an altered microbiota composition and reduced growth rates of the inner mucus layer along with increased permeability. Interestingly, the mucus layer was rescued after the administration of the fiber inulin or *Bifidobacterium longum.* 106

Fructose, mostly from high-fructose corn syrup used as an ingredient in many highly processed food products, is thought to be a strong contributor to NAFLD/NASH development and its increasing consumption in Western-type diets may be an underlying reason for the rapidly increasing incidence of these diseases.^107^,^108^ As evidenced from mouse models, a high-fructose diet alters gut microbiota with *Bacteroidetes* significantly decreased and *Proteobacteria* significantly increased. The High-fructose diet also reduces total SCFA production, increases intestinal epithelial barrier impairment, and causes inflammasome dysfunction.^109^ A high-fructose diet also reduces commensal bile salt hydrolase-expressing microbes and increases luminal concentrations of conjugated BAs and levels of resultant colitis.^110^

Furthermore, in murine models, high dietary cholesterol led to steatosis, steatohepatitis, fibrosis, and eventually HCC and increased populations of *Mucispirillum, Desulfovibrio, Anaerotruncus,* and *Desulfovibrionaceae,* while reducing *Bifidobacterium* and *Bacteroides*. Germ-free mice gavaged with stool from high-fat, high-calorie–fed mice showed increased hepatic lipid accumulation, inflammation, and cell proliferation, recapitulating the donor mice phenomes. In the same study, the authors extended these findings by also observing these microbial alterations in patients with hypercholesteremia.^111^

A high-fat diet (HFD) can lead to hepatic steatosis, cell injury, portal and lobular inflammation, and fibrosis along with increases in the abundance of *Firmicutes* in the murine gut. At the genus level, increases in the abundance of *Adercreutzia, Coprococcus, Dorea,* and *Ruminococcus* and decreased abundances of *Turicibacter* and *Anaeroplasma* have been observed, mimicking some alterations seen in patients with NAFLD.^112^ Mechanistically, an HFD may lead to altered microbiota composition, accumulation of BAs in the liver, and activation of the proto-oncogenic mTOR pathway in hepatocytes.^113^

A comprehensive study recently revealed the effects of an HFD in mice. 16S rDNA analysis showed that the abundances of *Lactobacillus* were increased, and those of *Clostridium* drastically decreased in the small intestine. Intestinal permeability was demonstrated to be significantly altered through a fluorescein isothiocyanate-dextran assay. Levels of conjugated taurocholic acid were significantly increased, and those of secondary BAs were decreased in the small intestine. Finally, immunohistochemistry indicated that the infiltration of LPS was significantly increased in the liver of HFD-fed mice coupled with increased accumulation of fat drops within the liver.^114^

A recent 16s rRNA sequencing study assessing gut populations of patients with cirrhosis (with or without HCC) found a unique microbial signature in the HCC-cirrhosis group with an overrepresentation of *Clostridium* and CF231 and reduced *Alphaproteobacteria* abundances compared with the patients with cirrhosis alone. They also found that dietary sugar was significantly associated with alterations in the microbiome of patients with HCC-cirrhosis with significant enrichment of gram-negative *Cloacibacillus.* 115

Studies on human cohorts and in animals have associated choline-deficient diets with NAFLD.^116^,^117^ On the contrary, other studies have shown that increased serum choline levels are associated with NASH.^81^,^118^ Choline, which is mainly obtained from the diet, can be metabolized by the gut bacteria to trimethylamine, which is further oxidized to TMAO in the liver.

These studies suggest that an unhealthy diet increases steatosis and alters gut microbiome profiles, promoting metabolic dysfunction and altering colonic barrier integrity, which allows the translocation of bacteria and their toxins to the liver, culminating in an inflammatory milieu. While the effect of diet on patients with liver disease is being investigated, more focus on the effect of diet on liver health mediated by the gut microbiome is still needed.

### Alcohol

The detrimental effects of excess alcohol intake on the gut microbiome were first documented over 30 years ago when bacterial overgrowth in the small intestine of patients with chronic alcohol abuse was observed.^119^ More recently, moderate alcohol consumption has also been associated with intestinal bacterial overgrowth.^120^

Analysis of the fecal metabolome revealed that the stool of patients with alcohol use disorder showed decreases in the SCFAs propionate and isobutyrate compared with controls. These are important metabolites for maintaining intestinal epithelial cell health and barrier integrity.^121^

A Norwegian metagenomic study of 24 excessive alcohol consumers compared with 18 controls observed that *Proteobacteria* species were increased while *Faecalibacterium* bacteria were decreased in the high intake group. They also found lower concentrations of butyrate in the high alcohol users, while other SCFA levels were similar between groups.^122^

In addition, a study investigated the effect of smoking and alcohol drinking on the gut microbiome. Analysis of 4 small groups of between 14 and 43 participants (nonsmoking and nondrinking, smoking only, drinking only, and smoking and drinking combined) showed that *Firmicutes* were reduced in the smoking and drinking groups, especially *Ruminococcaceae*, a producer of SCFAs. In concordance with this, SCFAs were reduced in patients with alcohol consumption.^123^

In a study on the effect of alcohol on the gut-liver axis, Kakiyama et al, assessed inflammation markers, intestinal barrier integrity, microbiota composition and functionality in a cohort of 103 patients, which included 19 controls, 6 drinkers without liver disease, and 78 patients with cirrhosis. They found increases in total and secondary BA concentrations in fecal samples of all current drinkers along with increases in proinflammatory cytokines TNFα, IL6, IL1β, MCP1, and COX2 in active patients with alcohol use disorder. Serum DCA and LPS levels were also seen to be elevated in current drinkers relative to abstainers.^124^

### Exercise

A systematic review and meta-analysis demonstrated the link between liver cancer risk and low levels of physical activity.^125^ Alpha diversity of the gut microbiome was increased in obese and overweight patients after a 3-month program of high-intensity exercise relative to sedentary controls, while changes were also reported in beta diversity in groups with high and moderate-intensity programs.^126^ However, the effect of exercise on the link between the gut microbiome and HBCs is poorly characterized and we are not aware of any human studies on how exercise impacts liver disease as mediated by the microbiome.

In an early onset obesity and NAFLD rat model, exercise increased *Parabacteroides, Bacteroides,* and *Flavobacterium* genera and reduced *Blautia*, *Dysgonomonas,* and *Porphyromonas*. These alterations were associated with changes in fecal BAs, Farnesoid *X* receptor expression, and circulating LPS, with indicators of improvements in metabolomic profiles and intestinal barrier functioning culminating in reduced steatosis as assessed by histology and reduced expression of hepatic and intestinal TLR4, TNFα, and IL6.^127^

A diet and exercise study in rats revealed that a regimen of *Lycium barbarum* polysaccharides, a potential prebiotic and aerobic exercise ameliorated NAFD. The intervention restored gut microbiota composition following HFD-induced dysbiosis, increased fecal SCFA concentrations, and restored the colonic and ileum tight junctions by increasing the expression of ZO-1 and occludin. Gut-derived LPS and related NF-κB signaling in the liver were also reduced as determined by immunohistochemistry and western blot.^128^

Further study is needed to elucidate the effect of exercise on the gut microbiome and the downstream effects on HBCs.

### Circadian rhythm

Circadian rhythm disruption is classified by the International Agency for Research on Cancer as a group 2A carcinogen.^129^ Specific taxa of the gut microbiome and their metabolic products oscillate over 24-hour periods. These diurnal compositional and functional oscillations affect metabolic homeostasis in mouse models with primary and secondary BAs displaying significant alterations in serum concentrations.^130^,^131^ Gut-derived SCFAs also display a diurnal rhythmicity that is disrupted by night shift work.^132^ This microbial oscillation has been seen to be regulated by the host circadian rhythm in mouse models, however, the microbiota diurnal rhythmicity also influences the host circadian rhythm.^130^,^133^

Circadian rhythm disruption has also been linked to liver disease development. This may be through alterations in melatonin secretion as animal studies suggest that melatonin may enhance gut microbiome diversity and reduce hepatic steatosis.^134^ Circadian disruption may also promote hepatic steatosis through changes in dietary and lifestyle patterns induced by altered sleep patterns.^135^ The liver functions as a peripheral circadian clock controlling and impacting the rhythmicity of hepatic metabolism.^136^ This liver circadian clock has been seen to be modified by diet, for example by high-fat or ketogenic dietary patterns but these data originate largely from experimental and animal models while human studies are sparse.^137^,^138^

## POTENTIAL THERAPEUTIC AND PREVENTATIVE AVENUES

Perturbation of intestinal flora composition and the gut-liver axis after certain dietary and lifestyle patterns affect the progression of hepatobiliary disease. In addition, the microbiome has been observed to alter responses to existing therapies. Immunotherapeutics have shown great promise in the treatment of HCC. A combination of atezolizumab and bevacizumab has been shown to improve overall survival leading to FDA approval of this regimen.^139^ Metagenomic analysis of fecal samples from anti-PD-1 immunotherapy responders had higher bacterial levels of *Akkermansia muciniphila* and *Ruminococcaceae* species while nonresponders had higher *Proteobacteria* levels.^140^ More recently, the fecal samples of patients with HCC who achieved disease control after treatment with tremelimumab and/or durvalumab had higher pretreatment relative abundances of *Akkermansia,* while those of *Enterobacteriaceae* were lower.^141^ Sorafenib is a tyrosine kinase inhibitor used in advanced HCC to reduce proliferation and angiogenesis. Increased *Butyricimonas*, *Veillonella, Bacillus*, *Enterobacter, Faecalibacterium, Lachnospira, Dialister,* and *Anaerostipes* have been found in the stool of patients displaying less adverse effects after treatment with sorafenib.^142^

These studies illustrate the potential utility of microbiome characteristics as biomarkers, which may allow for the development of more personalized HCC treatment regimens. Several avenues are being explored to balance the gut microbiota and restore gut barrier integrity, which may help improve hepatobiliary health and potentially increase the efficacy and tolerability of existing therapies.

### Antibiotics

In their seminal 2012 study in mice, Dapito et al, showed that ablation of the gut microbiome through a cocktail of ampicillin, vancomycin, neomycin, and metronidazole caused a reduction in liver tumor number, size, and liver-body weight ratio relative to healthy mice. However, this effect was not seen once carcinogenesis had occurred.^143^ Subsequently, other antibiotics have been investigated.

PSC is a chronic inflammatory liver disease and is frequent in cases of ulcerative colitis after epithelial barrier dysfunction.^144^ Recently, *Klebsiella pneumoniae* was identified as a culprit in PSC leading to bacterial translocation and TH17-mediated liver inflammation in mouse models. Treatment with metronidazole or vancomycin ameliorated TH17 responses in the liver highlighting the potential utility of microbiome editing using antibiotics.^145^

Rifaximin is an attractive antibiotic due to its high therapeutic index and broad-spectrum effects against gram-positive and gram-negative aerobes and anaerobes.^146^ It has low absorption after oral administration increasing suitability for targeting the gut microbiota. In obese mouse models, rifaximin was shown to prevent ethanol-induced liver injury through the modulation of small intestinal microbiota populations.^147^ A recent phase 4 randomized clinical trial concluded that low-dose rifaximin significantly decreases the occurrence of overall complications, leading to prolonged survival in patients with advanced stages of cirrhosis. The authors note that no effect was observed on the rate of tumorigenesis and speculate that this may be due to the short observation period. They recommend a longer study to assess any potential benefit for individuals at risk of progressing from cirrhosis to neoplasm.^148^

Similarly, patients with cirrhosis undergoing selective intestinal bacterial ablation with norfloxacin show a reduction in serum concentrations of pro-inflammatory cytokines TNFα, IFNγ, and IL12 and reduced activation of NF-Κb as measured by ELISA and western blot respectively.^149^

Targeting of gram-positive bacteria with vancomycin in primary and liver metastasis mouse models was observed to have an antitumor effect. Ablation of gram-positive bacteria, which perform the conversion of primary to secondary BAs, was sufficient to increase CXCL16 production and induce hepatic natural killer T cell infiltration and decrease liver tumor growth.^71^

### Probiotics

Probiotics are oral supplements of microorganism strains that are purported to confer health benefits through the competitive exclusion of pathogens, promoting a healthy balanced flora, and bolstering gut barrier and immune function.^150^ The mechanisms, by which probiotics may attenuate HCC development have been reviewed elsewhere but include bolstering the maintenance of gut epithelial barrier function, inhibiting translocation of gut bacteria and their products along with promoting the growth of bacteria producing anti-inflammatory metabolites. Furthermore, probiotics could ameliorate hepatic lipotoxicity and reduce the expression of angiogenic factors VEGFA and ANGPTs.^151^

In high-sugar–induced NAFLD mouse models, *Lactobacillus rhamnosus GG* has been seen to restore gut barrier function resulting in the attenuation of liver inflammation and steatosis,^152^ while in high-fat–induced murine NAFLD, *Lactobacillus rhamnosus GG* reduced host intestinal fatty acid absorption and accompanied hepatic steatosis.^153^ Furthermore, *Bifidobacterium*-treated rats were also seen to have reduced secretion of inflammatory cytokines and increased intestinal barrier proteins both in vitro and in vivo.^154^

Despite encouraging results in animal models and numerous studies on the role of probiotics in colorectal cancer, data from hepatobiliary disease clinical trials are less common.^155^ An 8-week randomized clinical trial involving diabetic patients with NAFLD administered a combination of 14 probiotic bacteria belonging to *Lactobacillus*, *Lactococcus*, *Bifidobacterium,* and *Propionibacterium* genera or a placebo. A reduction in fat deposition in the liver along with reduced serum concentrations of inflammatory markers TNFα IL6 and liver damage marker GGT was observed in the probiotic group.^156^

Another 6-week clinical trial suggested that supplementation with *Bacillus subtilis R0179* and *Bifidobacterium animalis subsp. lactis. B94* altered BA metabolism as increased plasma concentrations of deconjugated BAs were observed in obese individuals after probiotic administration.^157^ A multistrain probiotic mixture of several *Lactobacillus* and *Bifidobacterium* species and *Streptococcus thermophilus* was seen to improve the liver histology of patients with NAFLD by reducing hepatocyte ballooning and hepatic fibrosis. Reductions in hepatic inflammation and levels of alanine transaminase, adipocytokines, leptin, and endotoxins were also observed.^158^ The first trial in lean patients with NAFLD observed that a similar probiotic combination reduced inflammatory cytokines, hepatic steatosis, and fibrosis relative to controls.^159^

### Fecal microbiota transfer

Fecal microbiota transfer (FMT) is the transplant of a fecal suspension derived from a healthy donor to a patient to alter the bacterial composition of the recipient’s digestive tract. In murine models, ethanol-induced dysbiosis, steatosis, and liver inflammation were ameliorated after FMT and restoration of gut homeostasis.^160^

Similarly, FMT corrected dysbiosis in HFD-induced steatohepatitis in mice and increased gut butyrate concentrations, as measured by 16s sequencing and high-pressure liquid chromatography performed on cecal contents respectively. Following FMT, the expression of intestinal tight junction protein ZO-1 was also demonstrated to be significantly increased in the small intestine by immunohistochemistry and qPCR. Immunohistochemistry also revealed that intrahepatic IFNγ and IL17 were decreased after the intervention.^161^

In the clinic, in a small pilot study in patients with steroid-ineligible severe alcoholic hepatitis, FMT reduced disease severity and increased survival at one-year follow-up.^162^ FMT, using stool selected for the highest abundance of *Lachospiraceae* and *Ruminococcaceae*, was also seen to reduce hospitalizations, improve cognition and ameliorate dysbiosis in patients with cirrhosis and recurrent hepatic encephalopathy.^163^

Although the potential for the treatment of liver disease through microbiome modification is promising, concerns exist about the potential for the introduction of pathogenic species and bacteremia after bacterial translocation in immunocompromised patients. Some of these concerns might be circumvented by the use of lab-grown microbiota consortia which are currently in clinical trials to treat recurrent *C. difficile* infection.^164^

### Bacteriophages

It has long been recognized that abundant populations of nonpathogenic viruses, primarily bacteriophages, live in the mammalian gut and prey on resident bacteria.^165^ More recently, attempts have been made to harness their activity for therapeutic gain.

Duan and colleagues investigated cytolysin, a metabolite of *Enterococcus faecalis* and endotoxin that causes hepatocyte death. The presence of cytolysin-positive *E. faecalis* was also seen to correlate with liver disease severity and mortality in patients with alcoholic hepatitis. They then used 4 phages of the *Picovirinae* group to target cytolytic *E. faecalis* and abolished ethanol-induced liver disease in humanized mice.^166^

The complex phage community is a current gap in the literature and our understanding of the human microbiome and further study will be necessary before phages can be applied for reliable and consistent microbiome editing.

## FUTURE AVENUES

There is great promise in studying the microbiome in relation to hepatobiliary health, particularly in the areas of discovering molecular mechanisms, new biomarkers, therapies, and lifestyle interventions. The microbiome has the potential to be used as a noninvasive screening tool and prognostic indicator, predicting treatment response and paving the way for more personalized therapeutic modalities. However, many hurdles remain before this promise can be fully realized.

Much of the current literature arises from animal studies while the data on humans are predominantly from descriptive and cross-sectional analyses or small case-control studies. Case-control studies are typically reliant on samples taken at diagnosis allowing for the potential of reverse causation. Given the mailability of the gut microbiome in response to external factors, more data from carefully controlled randomized trials are also called for. There is also a need then for human mechanistic studies and increased integration of microbiome research into oncology studies and trials, along with data from prospective cohort studies where data and biospecimens are collected before disease diagnosis.^167^ Many large prospective cohorts have biobanked blood samples, but often lack stool and/or tissue sample collections and thus are limited in the ability to address hypotheses on the gut microbiome. Although some innovative data are arising from prospective cohorts linking specific microbiota species to cancer development,^168^ the application of blood-based analytical methods that proxy gut microbiome profiles and provide a more holistic view, such as plasma antibody repertoires, are required to move the field sufficiently forward.^169^

The literature also needs more data from diverse populations. Dietary and lifestyle patterns differ considerably between world regions, as does the incidence of liver diseases. It is highly likely that microbiome composition and its metabolic activity follow suit. Observed associations between microbiome profiles and liver diseases may therefore differ by population, but compelling data are currently lacking.

Reproducibility of results from metagenomic studies is affected by biospecimen type, geographical origin, DNA extraction protocol, contamination, and bioinformatic pipeline choices but standardization of these methods has proven difficult due to the diversity of the biological questions being investigated. However, standardization of sample collection and storage and the use of extensive positive and negative controls during analysis may prove helpful in reducing variation.^170^ The reproducibility of results is also hampered by the temporal oscillations of microbiome populations within individuals indicating a need for incorporation of repeated measurements into the study design although this may prove logistically challenging.^171^

The majority of published studies focus on the impact of dysbiosis on liver disease development. In doing so, they neglect an element of what is likely to be, to some degree, a bi-directional relationship in that liver disease development may alter gut microbiome composition and metabolic activity. This is exemplified by liver cholesterol metabolism and BA pathways that are altered with hepatitis.^172^ Alteration of BA secretion may modulate the nutrients reaching the gut and hence alter the microbiome towards an unfavorable profile, which in-turn could further negatively impact the health of the liver. In this regard, liver functionality can be conceptualized not only as a mediator but also as a modulator, and the role may change over time and with the degree of dysfunction, dysbiosis, and advancement of the disease.

To date, the majority of microbiome studies aiming to identify diagnostic and prognostic biomarkers have been one-dimensional, assessing only fecal bacteria populations. There is also a paucity of interventional randomized data on whether modulation of the gut microbiome towards a healthier profile can prevent or modulate liver disease development. There is a need for multidisciplinary studies akin to that conducted by Leung et al,^34^ which incorporated metagenomic, metabolomics, and clinical characteristics to predict NAFLD risk in a longitudinal study. There is also evidence from genome-wide association studies indicating an influence of human genetic variation on the composition of the microbiome adding another potential avenue of inquiry through methods such as Mendelian randomization.^173^ In the future, deeper mechanistic studies, such as investigating microbiome activity through metatranscriptomics and metabolic activity through metabolomics will be needed. Incorporation of multiomics data will come with statistical and computational challenges, inherent in analyzing multidimensional data, but will potentially yield greater insights into complex host-microbiome-disease interactions.

## CONCLUSION

This review highlights the emerging evidence suggesting that the gut microbiome is intrinsically tied to HCC development. The findings from the studies described here illustrate how alterations in intestinal bacterial abundances can result in increased translocation and altered BA synthesis. This can lead to altered immune and inflammatory factors in the liver contributing to liver disease and possibly neoplasia. Furthermore, lifestyle factors such as diet, alcohol, exercise, and circadian rhythm have been shown to alter the abundance of bacterial populations and by extension their metabolites, potentially impacting liver health. Data are emerging from the clinic on the utility of antibiotics and probiotics in NAFLD and NASH to target genotoxic or BA metabolizing species. However, investigation of other microbiome editing techniques, such as bacteriophages and FMT, is just beginning to enter the clinic based on the encouraging preclinical data. Finally, a multiomics approach, and more comprehensive data from large prospective studies, will be needed to fully realize the potential of microbiome studies in this area, elucidating the mechanistic underpinnings between the associations of the microbiota of the gut-liver axis with metabolic dysfunction, unhealthy dietary and lifestyle habits, obesity, and liver disease.

## References

[1] Rumgay H Arnold M Ferlay J Lesi O Cabasag CJ Vignat J . Global burden of primary liver cancer in 2020 and predictions to 2040. J Hepatol. 2022;77:1598–606. 36208844 10.1016/j.jhep.2022.08.021 PMC9670241

[2] Blumgart LH Fong Y . Hepatobiliary cancer. Semin Surg Oncol. 2000;19:83. 11135475 10.1002/1098-2388(200009)19:2<83::aid-ssu1>3.0.co;2-6

[3] IACR. Cancer Today. Accessed December 19, 2022. https://gco.iarc.fr/today/online-analysis-table?v=2020&mode=cancer&mode_population=continents&population=900&populations=900&key=asr&sex=0&cancer=39&type=1&statistic=5&prevalence=0&population_group=0&ages_group%5B%5D=0&ages_group%5B%5D=17&group_cancer=1&include_nmsc=1&include_nmsc_other=1.

[4] Llovet JM Zucman-Rossi J Pikarsky E Sangro B Schwartz M Sherman M . Hepatocellular carcinoma. Nat Rev Dis Primers. 2016;2:16018. 27158749 10.1038/nrdp.2016.18

[5] Razumilava N Gores GJ . Cholangiocarcinoma. Lancet. 2014;383:2168–79. 24581682 10.1016/S0140-6736(13)61903-0 PMC4069226

[6] Rao CV Asch AS Yamada HY . Frequently mutated genes/pathways and genomic instability as prevention targets in liver cancer. Carcinogenesis. 2016;38:2–11. 27838634 10.1093/carcin/bgw118 PMC5219050

[7] Schwabe RF Greten TF . Gut microbiome in HCC—mechanisms, diagnosis and therapy. J Hepatol. 2020;72:230–238. 31954488 10.1016/j.jhep.2019.08.016

[8] Mariat D Firmesse O Levenez F Guimarăes V Sokol H Doré J . The Firmicutes/Bacteroidetes ratio of the human microbiota changes with age. BMC Microbiol. 2009;9:123. 19508720 10.1186/1471-2180-9-123 PMC2702274

[9] DeGruttola AK Low D Mizoguchi A Mizoguchi E . Current understanding of dysbiosis in disease in human and animal models. Inflamm Bowel Dis. 2016;22:1137–50. 27070911 10.1097/MIB.0000000000000750 PMC4838534

[10] Aoun A Darwish F Hamod N . The influence of the gut microbiome on obesity in adults and the role of probiotics, prebiotics, and synbiotics for weight loss. Prev Nutr Food Sci. 2020;25:113–23. 32676461 10.3746/pnf.2020.25.2.113 PMC7333005

[11] Duttaroy AK . Role of gut microbiota and their metabolites on atherosclerosis, hypertension and human blood platelet function: a review. Nutrients. 2021;13:144. 33401598 10.3390/nu13010144 PMC7824497

[12] Cullin N Azevedo Antunes C Straussman R Stein-Thoeringer CK Elinav E . Microbiome and cancer. Cancer Cell. 2021;39:1317–41. 34506740 10.1016/j.ccell.2021.08.006

[13] Zeuzem S . Gut-liver axis. Int J Colorectal Dis. 2000;15:59–82. 10855547 10.1007/s003840050236

[14] Berg RD Garlington AW . Translocation of certain indigenous bacteria from the gastrointestinal tract to the mesenteric lymph nodes and other organs in a gnotobiotic mouse model. Infect Immun. 1979;23:403–11. 154474 10.1128/iai.23.2.403-411.1979 PMC414179

[15] Jang MH Sougawa N Tanaka T Hirata T Hiroi T Tohya K . CCR7 is critically important for migration of dendritic cells in intestinal lamina propria to mesenteric lymph nodes. J Immunol. 2006;176:803–10. 16393963 10.4049/jimmunol.176.2.803

[16] Balmer ML Slack E de Gottardi A Lawson MAE Hapfelmeier S Miele L . The liver may act as a firewall mediating mutualism between the host and its gut commensal microbiota. Sci Transl Med. 2014;6:237ra266. 10.1126/scitranslmed.3008618 24848256

[17] Seki E Tsutsui H Nakano H Tsuji NM Hoshino K Adachi O . Lipopolysaccharide-induced IL-18 secretion from murine Kupffer cells independently of myeloid differentiation factor 88 that is critically involved in induction of production of IL-12 and IL-1beta. J Immunol. 2001;166:2651–7. 11160328 10.4049/jimmunol.166.4.2651

[18] Kopydlowski KM Salkowski CA Cody MJ van Rooijen N Major J Hamilton TA . Regulation of macrophage chemokine expression by lipopolysaccharide in vitro and in vivo. J Immunol. 1999;163:1537–44. 10415057

[19] Seki E Schnabl B . Role of innate immunity and the microbiota in liver fibrosis: crosstalk between the liver and gut. J Physiol. 2012;590:447–58. 22124143 10.1113/jphysiol.2011.219691 PMC3379693

[20] Heymann F Tacke F . Immunology in the liver—from homeostasis to disease. Nat Rev Gastroenterol Hepatol. 2016;13:88–110. 26758786 10.1038/nrgastro.2015.200

[21] Wree A Broderick L Canbay A Hoffman HM Feldstein AE . From NAFLD to NASH to cirrhosis-new insights into disease mechanisms. Nat Rev Gastroenterol Hepatol. 2013;10:627–36. 23958599 10.1038/nrgastro.2013.149

[22] Alexopoulou A Agiasotelli D Vasilieva LE Dourakis SP . Bacterial translocation markers in liver cirrhosis. Ann Gastroenterol. 2017;30:486–97. 28845103 10.20524/aog.2017.0178 PMC5566768

[23] Behary J Raposo AE Amorim NML Zheng H Gong L McGovern E . Defining the temporal evolution of gut dysbiosis and inflammatory responses leading to hepatocellular carcinoma in Mdr2 −/− mouse model. BMC Microbiol. 2021;21:113. 33858327 10.1186/s12866-021-02171-9 PMC8048083

[24] Ren Z Li A Jiang J Zhou L Yu Z Lu H . Gut microbiome analysis as a tool towards targeted non-invasive biomarkers for early hepatocellular carcinoma. Gut. 2019;68:1014–23. 30045880 10.1136/gutjnl-2017-315084 PMC6580753

[25] Piñero F Vazquez M Baré P Rohr C Mendizabal M Sciara M . A different gut microbiome linked to inflammation found in cirrhotic patients with and without hepatocellular carcinoma. Ann Hepatol. 2019;18:480–7. 31023615 10.1016/j.aohep.2018.10.003

[26] Ponziani FR Bhoori S Castelli C Putignani L Rivoltini L Del Chierico F . Hepatocellular carcinoma is associated with gut microbiota profile and inflammation in nonalcoholic fatty liver disease. Hepatology. 2019;69:107–20. 29665135 10.1002/hep.30036

[27] Albhaisi S Shamsaddini A Fagan A McGeorge S Sikaroodi M Gavis E . Gut microbial signature of hepatocellular cancer in men with cirrhosis. Liver Transpl. 2021;27:629–40. 33492761 10.1002/lt.25994

[28] Behary J Amorim N Jiang X-T Raposo A Gong L McGovern E . Gut microbiota impact on the peripheral immune response in non-alcoholic fatty liver disease related hepatocellular carcinoma. Nat Commun. 2021;12:187. 33420074 10.1038/s41467-020-20422-7 PMC7794332

[29] Liu Q Li F Zhuang Y Xu J Wang J Mao X . Alteration in gut microbiota associated with hepatitis B and non-hepatitis virus related hepatocellular carcinoma. Gut Pathog. 2019;11:1. 30675188 10.1186/s13099-018-0281-6 PMC6337822

[30] Jia X Lu S Zeng Z Liu Q Dong Z Chen Y . Characterization of gut microbiota, bile acid metabolism, and cytokines in intrahepatic cholangiocarcinoma. Hepatology. 2020;71:893–906. 31298745 10.1002/hep.30852

[31] Hashimoto E Taniai M Tokushige K . Characteristics and diagnosis of NAFLD/NASH. J Gastroenterol Hepatol. 2013;28(suppl 4):64–70. 24251707 10.1111/jgh.12271

[32] Loomba R Seguritan V Li W Long T Klitgord N Bhatt A . Gut microbiome-based metagenomic signature for non-invasive detection of advanced fibrosis in human nonalcoholic fatty liver disease. Cell Metab. 2017;25:1054–62.e1055. 28467925 10.1016/j.cmet.2017.04.001 PMC5502730

[33] Oh TG Kim SM Caussy C Fu T Guo J Bassirian S . A universal gut-microbiome-derived signature predicts cirrhosis. Cell Metab. 2020;32:878–88.e876. 32610095 10.1016/j.cmet.2020.06.005 PMC7822714

[34] Leung H Long X Ni Y Qian L Nychas E Siliceo SL . Risk assessment with gut microbiome and metabolite markers in NAFLD development. Sci Translat Med. 2022;14:eabk0855. 10.1126/scitranslmed.abk0855 PMC9746350 35675435

[35] Wong VW-S Tse C-H Lam TT-Y Wong GLH Chim AML Chu WCW . Molecular characterization of the fecal microbiota in patients with nonalcoholic steatohepatitis--a longitudinal study. PLoS One. 2013;8:e62885. 23638162 10.1371/journal.pone.0062885 PMC3636208

[36] Boursier J Mueller O Barret M Machado M Fizanne L Araujo‐Perez F . The severity of nonalcoholic fatty liver disease is associated with gut dysbiosis and shift in the metabolic function of the gut microbiota. Hepatology. 2016;63:764–75. 26600078 10.1002/hep.28356 PMC4975935

[37] Day CP James OFW . Steatohepatitis: a tale of two “hits”? Gastroenterology. 1998;114:842–45. 9547102 10.1016/s0016-5085(98)70599-2

[38] Mutlu EA Gillevet PM Rangwala H Sikaroodi M Naqvi A Engen PA . Colonic microbiome is altered in alcoholism. Am J Physiol Gastrointest Liver Physiol. 2012;302:G966–78. 22241860 10.1152/ajpgi.00380.2011 PMC3362077

[39] Chen Y Yang F Lu H Wang B Chen Y Lei D . Characterization of fecal microbial communities in patients with liver cirrhosis. Hepatology. 2011;54:562–72. 21574172 10.1002/hep.24423

[40] Maccioni L Gao B Leclercq S Pirlot B Horsmans Y De Timary P . Intestinal permeability, microbial translocation, changes in duodenal and fecal microbiota, and their associations with alcoholic liver disease progression in humans. Gut Microbes. 2020;12:1782157. 32588725 10.1080/19490976.2020.1782157 PMC7524402

[41] Yang AM Inamine T Hochrath K Chen P Wang L Llorente C . Intestinal fungi contribute to development of alcoholic liver disease. J Clin Invest. 2017;127:2829–41. 28530644 10.1172/JCI90562 PMC5490775

[42] Deng T Li J He B Chen B Liu F Chen Z . Gut microbiome alteration as a diagnostic tool and associated with inflammatory response marker in primary liver cancer. Hepatol Int. 2022;16:99–111. 35064546 10.1007/s12072-021-10279-3

[43] Geng HW Yin FY Zhang ZF Gong X Yang Y . Butyrate suppresses glucose metabolism of colorectal cancer cells via GPR109a-AKT signaling pathway and enhances chemotherapy. Front Mol Biosci. 2021;8:634874. 33855046 10.3389/fmolb.2021.634874 PMC8039130

[44] Yang B Petrick JL Thistle JE Pinto LA Kemp TJ Tran HQ . Bacterial translocation and risk of liver cancer in a finnish cohort. Cancer Epidemiol Biomarkers Prev. 2019;28:807–13. 30602499 10.1158/1055-9965.EPI-18-0240 PMC7197395

[45] Fedirko V Tran HQ Gewirtz AT Stepien M Trichopoulou A Aleksandrova K . Exposure to bacterial products lipopolysaccharide and flagellin and hepatocellular carcinoma: a nested case-control study. BMC Med. 2017;15:72. 28372583 10.1186/s12916-017-0830-8 PMC5379669

[46] Darnaud M Faivre J Moniaux N . Targeting gut flora to prevent progression of hepatocellular carcinoma. J Hepatol. 2013;58:385–7. 22940407 10.1016/j.jhep.2012.08.019

[47] Parada Venegas D De la Fuente MK Landskron G González MJ Quera R Dijkstra G . Short chain fatty acids (SCFAs)-mediated gut epithelial and immune regulation and its relevance for inflammatory bowel diseases. Front Immunol. 2019;10:277. 30915065 10.3389/fimmu.2019.00277 PMC6421268

[48] Petrick JL Florio AA Zen J Wang Y Gewirtz AT Pfeiffer RM . Biomarkers of gut barrier dysfunction and risk of hepatocellular carcinoma in the REVEAL-HBV and REVEALHCV cohort studies. Int J Cancer. 2023;153:44–53. 36878686 10.1002/ijc.34492 PMC10548479

[49] Lazaridis KN Gores GJ . Cholangiocarcinoma. Gastroenterology. 2005;128:1655–67. 15887157 10.1053/j.gastro.2005.03.040

[50] Chng KR Chan SH Ng AHQ Li C Jusakul A Bertrand D . Tissue microbiome profiling identifies an enrichment of specific enteric bacteria in opisthorchis viverrini associated cholangiocarcinoma. EBioMedicine. 2016;8:195–202. 27428430 10.1016/j.ebiom.2016.04.034 PMC4919562

[51] Williamson KD Chapman RW . Primary sclerosing cholangitis. Dig Dis. 2014;32:438–45. 24969292 10.1159/000358150

[52] Zhang Q Ma C Duan Y Heinrich B Rosato U Diggs LP . Gut microbiome directs hepatocytes to recruit MDSCs and promote cholangiocarcinoma. Cancer Discov. 2021;11:1248–67. 33323397 10.1158/2159-8290.CD-20-0304 PMC8102309

[53] Jia W Xie G Jia W . Bile acid–microbiota crosstalk in gastrointestinal inflammation and carcinogenesis. Nature Rev Gastroenterol Hepatol. 2018;15:111–28. 29018272 10.1038/nrgastro.2017.119 PMC5899973

[54] Cook JW Kennaway EL Kennaway NM . Production of tumours in mice by deoxycholic acid. Nature. 1940;145:627.

[55] Šarenac TM Mikov M . Bile acid synthesis: from nature to the chemical modification and synthesis and their applications as drugs and nutrients. Front Pharmacol. 2018;9:939. 30319399 10.3389/fphar.2018.00939 PMC6168039

[56] Foley MH O’Flaherty S Barrangou R Theriot CM . Bile salt hydrolases: gatekeepers of bile acid metabolism and host-microbiome crosstalk in the gastrointestinal tract. PLoS Pathog. 2019;15:e1007581. 30845232 10.1371/journal.ppat.1007581 PMC6405046

[57] Chiang JYL . Bile acids: regulation of synthesis. J Lipid Res. 2009;50:1955–66. 19346330 10.1194/jlr.R900010-JLR200 PMC2739756

[58] Wang H Chen J Hollister K Sowers LC Forman BM . Endogenous bile acids are ligands for the nuclear receptor FXR/BAR. Mol Cell. 1999;3:543–53. 10360171 10.1016/s1097-2765(00)80348-2

[59] Maruyama T Miyamoto Y Nakamura T Tamai Y Okada H Sugiyama E . Identification of membrane-type receptor for bile acids (M-BAR). Biochem Biophys Res Commun. 2002;298:714–9. 12419312 10.1016/s0006-291x(02)02550-0

[60] Mouzaki M Wang AY Bandsma R Comelli EM Arendt BM Zhang L . Bile Acids and dysbiosis in non-alcoholic fatty liver disease. PLoS One. 2016;11:e0151829. 27203081 10.1371/journal.pone.0151829 PMC4874546

[61] Cao H Xu M Dong W Deng B Wang S Zhang Y . Secondary bile acid-induced dysbiosis promotes intestinal carcinogenesis. Int J Cancer. 2017;140:2545–56. 28187526 10.1002/ijc.30643

[62] Jusakul A Khuntikeo N Haigh WG Kuver R Ioannou GN Loilome W . Identification of biliary bile acids in patients with benign biliary diseases, hepatocellular carcinoma and cholangiocarcinoma. Asian Pac J Cancer Prev. 2012;13:77–82. 23480749

[63] Shukla VK Tiwari SC Roy SK . Biliary bile acids in cholelithiasis and carcinoma of the gall bladder. Eur J Cancer Prev. 1993;2:155–60. 8461866 10.1097/00008469-199303000-00008

[64] Dai J Wang H Shi Y Dong Y Zhang Y Wang J . Impact of bile acids on the growth of human cholangiocarcinoma via FXR. J Hematol Oncol. 2011;4:41. 21988803 10.1186/1756-8722-4-41 PMC3207959

[65] Wang W Yin X Li G Yi J Wang J . Expressions of farnesoid X receptor and myeloid cell leukemia sequence 1 protein are associated with poor prognosis in patients with gallbladder cancer. Chin Med J (Engl). 2014;127:2637–42. 25043081

[66] Su H Ma C Liu J Li N Gao M Huang A . Downregulation of nuclear receptor FXR is associated with multiple malignant clinicopathological characteristics in human hepatocellular carcinoma. Am J Physiol Gastrointest Liver Physiol. 2012;303:G1245–53. 23042943 10.1152/ajpgi.00439.2011 PMC3532459

[67] Nguyen PT Kanno K Pham QT Kikuchi Y Kakimoto M Kobayashi T . Senescent hepatic stellate cells caused by deoxycholic acid modulates malignant behavior of hepatocellular carcinoma. J Cancer Res Clin Oncol. 2020;146:3255–68. 32870388 10.1007/s00432-020-03374-9 PMC11804495

[68] Faget DV Ren Q Stewart SA . Unmasking senescence: context-dependent effects of SASP in cancer. Nat Rev Cancer. 2019;19:439–53. 31235879 10.1038/s41568-019-0156-2

[69] Loo TM Kamachi F Watanabe Y Yoshimoto S Kanda H Arai Y . Gut microbiota promotes obesity-associated liver cancer through PGE2-mediated suppression of antitumor immunity. Cancer Discov. 2017;7:522–38. 28202625 10.1158/2159-8290.CD-16-0932

[70] Liu B Qu L Yan S . Cyclooxygenase-2 promotes tumor growth and suppresses tumor immunity. Cancer Cell Int. 2015;15:106. 26549987 10.1186/s12935-015-0260-7 PMC4635545

[71] Ma C Han M Heinrich B Fu Q Zhang Q Sandhu M . Gut microbiome-mediated bile acid metabolism regulates liver cancer via NKT cells. Science. 2018;360:eaan5931. 29798856 10.1126/science.aan5931 PMC6407885

[72] Yoshimoto S Loo TM Atarashi K Kanda H Sato S Oyadomari S . Obesity-induced gut microbial metabolite promotes liver cancer through senescence secretome. Nature. 2013;499:97–101. 23803760 10.1038/nature12347

[73] Loftfield E Rothwell JA Sinha R Keski-Rahkonen P Robinot N Albanes D . Prospective investigation of serum metabolites, coffee drinking, liver cancer incidence, and liver disease mortality. J Natl Cancer Inst. 2020;112:286–94. 31168595 10.1093/jnci/djz122 PMC7073908

[74] Stepien M Keski‐Rahkonen P Kiss A Robinot N Duarte‐Salles T Murphy N . Metabolic perturbations prior to hepatocellular carcinoma diagnosis: Findings from a prospective observational cohort study. Int J Cancer. 2021;148:609–25. 32734650 10.1002/ijc.33236

[75] Petrick JL Florio AA Koshiol J Pfeiffer RM Yang B Yu K . Prediagnostic concentrations of circulating bile acids and hepatocellular carcinoma risk: REVEAL-HBV and HCV studies. Int J Cancer. 2020;147:2743–53. 32406072 10.1002/ijc.33051 PMC7529994

[76] Thomas CE Luu HN Wang R Xie G Adams-Haduch J Jin A . Association between pre-diagnostic serum bile acids and hepatocellular carcinoma: the Singapore Chinese Health Study. Cancers (Basel). 2021;13:2648. 34071196 10.3390/cancers13112648 PMC8198655

[77] Stepien M Lopez‐Nogueroles M Lahoz A Kühn T Perlemuter G Voican C . Prediagnostic alterations in circulating bile acid profiles in the development of hepatocellular carcinoma. Int J Cancer. 2022;150:1255–68. 34843121 10.1002/ijc.33885

[78] Lin R Zhan M Yang L Wang H Shen H Huang S . Deoxycholic acid modulates the progression of gallbladder cancer through N(6)-methyladenosine-dependent microRNA maturation. Oncogene. 2020;39:4983–5000. 32514152 10.1038/s41388-020-1349-6 PMC7314665

[79] Zhu L Baker SS Gill C Liu W Alkhouri R Baker RD . Characterization of gut microbiomes in nonalcoholic steatohepatitis (NASH) patients: a connection between endogenous alcohol and NASH. Hepatology. 2013;57:601–9. 23055155 10.1002/hep.26093

[80] Chen Y Liu Y Zhou R Chen X Wang C Tan X . Associations of gut-flora-dependent metabolite trimethylamine-N-oxide, betaine and choline with non-alcoholic fatty liver disease in adults. Sci Rep. 2016;6:19076. 26743949 10.1038/srep19076 PMC4705470

[81] León-Mimila P Villamil-Ramírez H Li XS Shih DM Hui ST Ocampo-Medina E . Trimethylamine N-oxide levels are associated with NASH in obese subjects with type 2 diabetes. Diabetes Metab. 2021;47:101183. 32791310 10.1016/j.diabet.2020.07.010 PMC8018562

[82] Zhao M Zhao L Xiong X He Y Huang W Liu Z . TMAVA, a metabolite of intestinal microbes, is increased in plasma from patients with liver steatosis, inhibits γ-butyrobetaine hydroxylase, and exacerbates fatty liver in mice. Gastroenterology. 2020;158:2266–81.e2227. 32105727 10.1053/j.gastro.2020.02.033

[83] Knudsen C Neyrinck AM Lanthier N Delzenne NM . Microbiota and nonalcoholic fatty liver disease: promising prospects for clinical interventions? Curr Opin Clin Nutr Metab Care. 2019;22:393–400. 31219825 10.1097/MCO.0000000000000584

[84] Roager HM Licht TR . Microbial tryptophan catabolites in health and disease. Nat Commun. 2018;9:3294. 30120222 10.1038/s41467-018-05470-4 PMC6098093

[85] Muto Y Sato S Watanabe A . Overweight and obesity increase the risk for liver cancer in patients with liver cirrhosis and long-term oral supplementation with branched-chain amino acid granules inhibits liver carcinogenesis in heavier patients with liver cirrhosis. Hepatol Res. 2006;35:204–14. 16737844 10.1016/j.hepres.2006.04.007

[86] Xie C Halegoua-DeMarzio D . Role of probiotics in non-alcoholic fatty liver disease: does gut microbiota matter? Nutrients. 2019;11:2837. 31752378 10.3390/nu11112837 PMC6893593

[87] Hoyles L Fernández-Real JM Federici M Serino M Abbott J Charpentier J . Molecular phenomics and metagenomics of hepatic steatosis in non-diabetic obese women. Nat Med. 2018;24:1070–80. 29942096 10.1038/s41591-018-0061-3 PMC6140997

[88] Agus A Planchais J Sokol H . Gut microbiota regulation of tryptophan metabolism in health and disease. Cell Host Microbe. 2018;23:716–724. 29902437 10.1016/j.chom.2018.05.003

[89] Sehgal R Ilha M Vaittinen M Kaminska D Männistö V Kärjä V . Indole-3-propionic acid, a gut-derived tryptophan metabolite, associates with hepatic fibrosis. Nutrients. 2021;13:3509. 34684510 10.3390/nu13103509 PMC8538297

[90] Krishnan S Ding Y Saedi N Choi M Sridharan GV Sherr DH . Gut microbiota-derived tryptophan metabolites modulate inflammatory response in hepatocytes and macrophages. Cell Rep. 2018;23:1099–111. 29694888 10.1016/j.celrep.2018.03.109 PMC6392449

[91] Ji Y Yin Y Sun L Zhang W . The molecular and mechanistic insights based on gut-liver axis: nutritional target for non-alcoholic fatty liver disease (nafld) improvement. Int J Mol Sci. 2020;21:3066. 32357561 10.3390/ijms21093066 PMC7247681

[92] Nicholson JK Holmes E Kinross J Burcelin R Gibson G Jia W . Host-gut microbiota metabolic interactions. Science. 2012;336:1262–7. 22674330 10.1126/science.1223813

[93] Ni Y Ni L Zhuge F Fu Z . The gut microbiota and its metabolites, novel targets for treating and preventing non-alcoholic fatty liver disease. Mol Nutr Food Res. 2020;64:e2000375. 32738185 10.1002/mnfr.202000375

[94] Usami M . Gut microbiota and host metabolism in liver cirrhosis. World J Gastroenterol. 2015;21:11597–608. 26556989 10.3748/wjg.v21.i41.11597 PMC4631963

[95] Kaliannan K Wang B Li XY Kim KJ Kang JX . A host-microbiome interaction mediates the opposing effects of omega-6 and omega-3 fatty acids on metabolic endotoxemia. Sci Rep. 2015;5:11276. 26062993 10.1038/srep11276 PMC4650612

[96] Generoso SV Rodrigues NM Trindade LM Paiva NC Cardoso VN Carneiro CM . Dietary supplementation with omega-3 fatty acid attenuates 5-fluorouracil induced mucositis in mice. Lipids Health Dis. 2015;14:54. 26063053 10.1186/s12944-015-0052-z PMC4473827

[97] Xiao G Tang L Yuan F Zhu W Zhang S Liu Z . Eicosapentaenoic acid enhances heat stress-impaired intestinal epithelial barrier function in Caco-2 cells. PLoS ONE. 2013;8:e73571. 24066055 10.1371/journal.pone.0073571 PMC3774713

[98] Gao X Liu X Xu J Xue C Xue Y Wang Y . Dietary trimethylamine N-oxide exacerbates impaired glucose tolerance in mice fed a high fat diet. J Biosci Bioeng. 2014;118:476–81. 24721123 10.1016/j.jbiosc.2014.03.001

[99] Barrea L Annunziata G Muscogiuri G Di Somma C Laudisio D Maisto M . Trimethylamine-N-oxide (TMAO) as novel potential biomarker of early predictors of metabolic syndrome. Nutrients. 2018;10:1971. 30551613 10.3390/nu10121971 PMC6316855

[100] Yuan J Chen C Cui J Lu J Yan C Wei X . Fatty liver disease caused by high-alcohol-producing Klebsiella pneumoniae. Cell Metab. 2019;30:675–88.e677. 31543403 10.1016/j.cmet.2019.08.018

[101] Engstler AJ Aumiller T Degen C Dürr M Weiss E Maier IB . Insulin resistance alters hepatic ethanol metabolism: studies in mice and children with non-alcoholic fatty liver disease. Gut. 2016;65:1564–71. 26006114 10.1136/gutjnl-2014-308379

[102] Camilleri M Lyle BJ Madsen KL Sonnenburg J Verbeke K Wu GD . Role for diet in normal gut barrier function: developing guidance within the framework of food-labeling regulations. Am J Physiol Gastrointest Liver Physiol. 2019;317:G17–39. 31125257 10.1152/ajpgi.00063.2019 PMC6689735

[103] George ES Sood S Broughton A Cogan G Hickey M Chan WS . The Association between diet and hepatocellular carcinoma: a systematic review. Nutrients. 2021;13:172. 33430001 10.3390/nu13010172 PMC7826815

[104] Martinez KB Leone V Chang EB . Western diets, gut dysbiosis, and metabolic diseases: Are they linked? Gut Microbes. 2017;8:130–42. 28059614 10.1080/19490976.2016.1270811 PMC5390820

[105] Desai MS Seekatz AM Koropatkin NM Kamada N Hickey CA Wolter M . A dietary fiber-deprived gut microbiota degrades the colonic mucus barrier and enhances pathogen susceptibility. Cell. 2016;167:1339–53 e1321. 27863247 10.1016/j.cell.2016.10.043 PMC5131798

[106] Schroeder BO Birchenough GMH Ståhlman M Arike L Johansson MEV Hansson GC . Bifidobacteria or fiber protects against diet-induced microbiota-mediated colonic mucus deterioration. Cell Host Microbe. 2018;23:27–40 e27. 29276171 10.1016/j.chom.2017.11.004 PMC5764785

[107] Jegatheesan P De Bandt JP . Fructose and NAFLD: the multifaceted aspects of fructose metabolism. Nutrients. 2017;9:230.28273805

[108] Roeb E Weiskirchen R . Fructose and non-alcoholic steatohepatitis. Front Pharmacol. 2021;12:634344. 33628193 10.3389/fphar.2021.634344 PMC7898239

[109] Li J-M Yu R Zhang L-P Wen SY Wang SJ Zhang XY . Dietary fructose-induced gut dysbiosis promotes mouse hippocampal neuroinflammation: a benefit of short-chain fatty acids. Microbiome. 2019;7:98. 31255176 10.1186/s40168-019-0713-7 PMC6599330

[110] Montrose DC Nishiguchi R Basu S Staab HA Zhou XK Wang H . Dietary fructose alters the composition, localization, and metabolism of gut microbiota in association with worsening colitis. Cell Mol Gastroenterol Hepatol. 2021;11:525–50. 32961355 10.1016/j.jcmgh.2020.09.008 PMC7797369

[111] Zhang X Coker OO Chu ES Fu K Lau HCH Wang YX . Dietary cholesterol drives fatty liver-associated liver cancer by modulating gut microbiota and metabolites. Gut. 2021;70:761–774. 32694178 10.1136/gutjnl-2019-319664 PMC7948195

[112] Velázquez KT Enos RT Bader JE Sougiannis AT Carson MS Chatzistamou I . Prolonged high-fat-diet feeding promotes non-alcoholic fatty liver disease and alters gut microbiota in mice. World J Hepatol. 2019;11:619–37. 31528245 10.4254/wjh.v11.i8.619 PMC6717713

[113] Yamada S Takashina Y Watanabe M Nagamine R Saito Y Kamada N . Bile acid metabolism regulated by the gut microbiota promotes non-alcoholic steatohepatitis-associated hepatocellular carcinoma in mice. Oncotarget. 2018;9:9925–39. 29515780 10.18632/oncotarget.24066 PMC5839411

[114] Nakanishi T Fukui H Wang X Nishiumi S Yokota H Makizaki Y . Effect of a high-fat diet on the small-intestinal environment and mucosal integrity in the gut-liver axis. Cells. 2021;10:3168. 34831391 10.3390/cells10113168 PMC8622719

[115] Lapidot Y Amir A Nosenko R Uzan-Yulzari A Veitsman E Cohen-Ezra O . Alterations in the Gut Microbiome in the Progression of Cirrhosis to Hepatocellular Carcinoma. mSystems. 2020;5:e00153–00120. 32546668 10.1128/mSystems.00153-20 PMC7300357

[116] Buchman AL Ament ME Sohel M Dubin M Jenden DJ Roch M . Choline deficiency causes reversible hepatic abnormalities in patients receiving parenteral nutrition: proof of a human choline requirement: a placebo-controlled trial. JPEN J Parenter Enteral Nutr. 2001;25:260–8. 11531217 10.1177/0148607101025005260

[117] Takahashi Y . Animal models of nonalcoholic fatty liver disease/nonalcoholic steatohepatitis. World J Gastroenterol. 2012;18:2300–8. 22654421 10.3748/wjg.v18.i19.2300 PMC3353364

[118] Imajo K Fujita K Yoneda M Shinohara Y Suzuki K Mawatari H . Plasma free choline is a novel non-invasive biomarker for early-stage non-alcoholic steatohepatitis: a multi-center validation study. Hepatol Res. 2012;42:757–66. 22780848 10.1111/j.1872-034X.2012.00976.x

[119] Bode JC Bode C Heidelbach R Dürr HK Martini GA . Jejunal microflora in patients with chronic alcohol abuse. Hepatogastroenterology. 1984;31:30–4. 6698486

[120] Gabbard SL Lacy BE Levine GM Crowell MD . The impact of alcohol consumption and cholecystectomy on small intestinal bacterial overgrowth. Dig Dis Sci. 2014;59:638–44. 24323179 10.1007/s10620-013-2960-y

[121] Couch RD Dailey A Zaidi F Navarro K Forsyth CB Mutlu E . Alcohol induced alterations to the human fecal VOC metabolome. PLoS One. 2015;10:e0119362. 25751150 10.1371/journal.pone.0119362 PMC4353727

[122] Bjørkhaug ST Aanes H Neupane SP Bramness JG Malvik S Henriksen C . Characterization of gut microbiota composition and functions in patients with chronic alcohol overconsumption. Gut Microbes. 2019;10:663–75. 30894059 10.1080/19490976.2019.1580097 PMC6866679

[123] Lin R Zhang Y Chen L Qi Y He J Hu M . The effects of cigarettes and alcohol on intestinal microbiota in healthy men. J Microbiol. 2020;58:926–37. 32997305 10.1007/s12275-020-0006-7

[124] Kakiyama G Hylemon PB Zhou H Pandak WM Heuman DM Kang DJ . Colonic inflammation and secondary bile acids in alcoholic cirrhosis. Am J Physiol Gastrointest Liver Physiol. 2014;306:G929–37. 24699327 10.1152/ajpgi.00315.2013 PMC4152166

[125] Lee J . Associations between physical activity and liver cancer risks and mortality: a systematic review and meta-analysis. Int J Environ Res Public Health. 2020;17:8943. 33271947 10.3390/ijerph17238943 PMC7730643

[126] Kern T Blond MB Hansen TH Rosenkilde M Quist JS Gram AS . Structured exercise alters the gut microbiota in humans with overweight and obesity-A randomized controlled trial. Int J Obes (Lond). 2020;44:125–35. 31467422 10.1038/s41366-019-0440-y

[127] Carbajo-Pescador S Porras D García-Mediavilla MV . Beneficial effects of exercise on gut microbiota functionality and barrier integrity, and gut-liver crosstalk in an in vivo model of early obesity and non-alcoholic fatty liver disease. Dis Model Mech. 2019;12:dmm039206. 30971408 10.1242/dmm.039206 PMC6550047

[128] Gao L-L Ma J-M Fan Y-N Zhang YN Ge R Tao XJ . Lycium barbarum polysaccharide combined with aerobic exercise ameliorated nonalcoholic fatty liver disease through restoring gut microbiota, intestinal barrier and inhibiting hepatic inflammation. Int J Biol Macromol. 2021;183:1379–92. 33992651 10.1016/j.ijbiomac.2021.05.066

[129] IARC. Accessed January 20, 2022 https://monographs.iarc.who.int/list-of-classifications .

[130] Thaiss CA Levy M Korem T Dohnalová L Shapiro H Jaitin DA . Microbiota diurnal rhythmicity programs host transcriptome oscillations. Cell. 2016;167:1495–510 e1412. 27912059 10.1016/j.cell.2016.11.003

[131] Zhang YKJ Guo GL Klaassen CD . Diurnal variations of mouse plasma and hepatic bile acid concentrations as well as expression of biosynthetic enzymes and transporters. PLoS One. 2011;6:e16683. 21346810 10.1371/journal.pone.0016683 PMC3035620

[132] Swanson GR Siskin J Gorenz A Shaikh M Raeisi S Fogg L . Disrupted diurnal oscillation of gut-derived Short chain fatty acids in shift workers drinking alcohol: Possible mechanism for loss of resiliency of intestinal barrier in disrupted circadian host. Transl Res. 2020;221:97–109. 32376406 10.1016/j.trsl.2020.04.004 PMC8136245

[133] Liang X Bushman FD FitzGerald GA . Rhythmicity of the intestinal microbiota is regulated by gender and the host circadian clock. Proc Natl Acad Sci U S A. 2015;112:10479–84. 26240359 10.1073/pnas.1501305112 PMC4547234

[134] Yin J Li Y Han H Chen S Gao J Liu G . Melatonin reprogramming of gut microbiota improves lipid dysmetabolism in high-fat diet-fed mice. J Pineal Res. 2018;65:e12524. 30230594 10.1111/jpi.12524

[135] Sato K Meng F Francis H Wu N Chen L Kennedy L . Melatonin and circadian rhythms in liver diseases: functional roles and potential therapies. J Pineal Res. 2020;68:e12639. 32061110 10.1111/jpi.12639 PMC8682809

[136] Crespo M Leiva M Sabio G . Circadian clock and liver cancer. Cancers (Basel). 2021;13:3631. 34298842 10.3390/cancers13143631 PMC8306099

[137] Eckel-Mahan KL Patel VR de Mateo S Orozco-Solis R Ceglia NJ Sahar S . Reprogramming of the circadian clock by nutritional challenge. Cell. 2013;155:1464–78. 24360271 10.1016/j.cell.2013.11.034 PMC4573395

[138] Tognini P Murakami M Liu Y Eckel-Mahan KL Newman JC Verdin E . Distinct Circadian Signatures in Liver and Gut Clocks Revealed by Ketogenic Diet. Cell Metab. 2017;26:523–38.e525. 28877456 10.1016/j.cmet.2017.08.015

[139] Llovet JM Castet F Heikenwalder M Maini MK Mazzaferro V Pinato DJ . Immunotherapies for hepatocellular carcinoma. Nat Rev Clin Oncol. 2022;19:151–72. 34764464 10.1038/s41571-021-00573-2

[140] Zheng Y Wang T Tu X Huang Y Zhang H Tan D . Gut microbiome affects the response to anti-PD-1 immunotherapy in patients with hepatocellular carcinoma. J Immunother Cancer. 2019;7:193. 31337439 10.1186/s40425-019-0650-9 PMC6651993

[141] Ponziani FR De Luca A Picca A Marzetti E Petito V Del Chierico F . Gut Dysbiosis and Fecal Calprotectin Predict Response to Immune Checkpoint Inhibitors in Patients With Hepatocellular Carcinoma. Hepatol Commun. 2022;6:1492–501. 35261212 10.1002/hep4.1905 PMC9134810

[142] Yamamoto K Kuzuya T Honda T Ito T Ishizu Y Nakamura M . Relationship Between Adverse Events and Microbiomes in Advanced Hepatocellular Carcinoma Patients Treated With Sorafenib. Anticancer Res. 2020;40:665–76. 32014907 10.21873/anticanres.13996

[143] Dapito DH Mencin A Gwak GY Pradere JP Jang MK Mederacke I . Promotion of hepatocellular carcinoma by the intestinal microbiota and TLR4. Cancer Cell. 2012;21:504–16. 22516259 10.1016/j.ccr.2012.02.007 PMC3332000

[144] O’Hara SP LaRusso NF . The gut-liver axis in primary sclerosing cholangitis: are pathobionts the missing link? Hepatology. 2019;70:1058–60. 31013359 10.1002/hep.30673 PMC6717517

[145] Nakamoto N Sasaki N Aoki R Miyamoto K Suda W Teratani T . Gut pathobionts underlie intestinal barrier dysfunction and liver T helper 17 cell immune response in primary sclerosing cholangitis. Nat Microbiol. 2019;4:492–503. 30643240 10.1038/s41564-018-0333-1

[146] Calanni F Renzulli C Barbanti M Viscomi GC . Rifaximin: beyond the traditional antibiotic activity. J Antibiot (Tokyo). 2014;67:667–70. 25095806 10.1038/ja.2014.106

[147] Kitagawa R Kon K Uchiyama A Arai K Yamashina S Kuwahara-Arai K . Rifaximin prevents ethanol-induced liver injury in obese KK-A(y) mice through modulation of small intestinal microbiota signature. Am J Physiol Gastrointest Liver Physiol. 2019;317:G707–15. 31509430 10.1152/ajpgi.00372.2018

[148] Zeng X Sheng X Wang PQ Xin HG Guo YB Lin Y . Low-dose rifaximin prevents complications and improves survival in patients with decompensated liver cirrhosis. Hepatol Int. 2021;15:155–65. 33385299 10.1007/s12072-020-10117-y

[149] Zapater P Caño R Llanos L Ruiz–Alcaraz AJ Pascual S Barquero C . Norfloxacin modulates the inflammatory response and directly affects neutrophils in patients with decompensated cirrhosis. Gastroenterology. 2009;137:1669–79 e1661. 19660462 10.1053/j.gastro.2009.07.058

[150] Woo J Ahn J . Probiotic-mediated competition, exclusion and displacement in biofilm formation by food-borne pathogens. Lett Appl Microbiol. 2013;56:307–13. 23362863 10.1111/lam.12051

[151] Thilakarathna WPDW Rupasinghe HPV Ridgway ND . Mechanisms by which probiotic bacteria attenuate the risk of hepatocellular carcinoma. Int J Mol Sci. 2021;22:2606. 33807605 10.3390/ijms22052606 PMC7961993

[152] Ritze Y Bárdos G Claus A Ehrmann V Bergheim I Schwiertz A . Lactobacillus rhamnosus GG protects against non-alcoholic fatty liver disease in mice. PLoS ONE. 2014;9:e80169. 24475018 10.1371/journal.pone.0080169 PMC3903470

[153] Jang E Kim UK Jang K Song YS Cha JY Yi H . A protective mechanism of probiotic Lactobacillus against hepatic steatosis via reducing host intestinal fatty acid absorption. Exp Mol Med. 2019;51:1–14. 10.1038/s12276-019-0293-4 PMC6802638 31409765

[154] Ling X Linglong P Weixia D Hong W . Protective effects of bifidobacterium on intestinal barrier function in lps-induced enterocyte barrier injury of Caco-2 monolayers and in a rat NEC model. PLoS One. 2016;11:e0161635. 27551722 10.1371/journal.pone.0161635 PMC4995054

[155] Drago L . Probiotics and colon cancer. Microorganisms. 2019;7:66. 30823471 10.3390/microorganisms7030066 PMC6463067

[156] Kobyliak N Abenavoli L Mykhalchyshyn G Kononenko L Boccuto L Kyriienko D . A multi-strain probiotic reduces the fatty liver index, cytokines and aminotransferase levels in NAFLD patients: evidence from a randomized clinical trial. J Gastrointestin Liver Dis. 2018;27:41–9. 29557414 10.15403/jgld.2014.1121.271.kby

[157] Culpepper T Rowe CC Rusch CT Burns AM Federico AP Girard SA . Three probiotic strains exert different effects on plasma bile acid profiles in healthy obese adults: randomised, double-blind placebo-controlled crossover study. Benef Microbes. 2019;10:497–509. 31090458 10.3920/BM2018.0151

[158] Duseja A Acharya SK Mehta M Chhabra S Rana S Das A . High potency multistrain probiotic improves liver histology in non-alcoholic fatty liver disease (NAFLD): a randomised, double-blind, proof of concept study. BMJ Open Gastroenterol. 2019;6:e000315. 10.1136/bmjgast-2019-000315 PMC6688701 31423319

[159] Mofidi F Poustchi H Yari Z Nourinayyer B Merat S Sharafkhah M . Synbiotic supplementation in lean patients with non-alcoholic fatty liver disease: a pilot, randomised, double-blind, placebo-controlled, clinical trial. Br J Nutr. 2017;117:662–68. 28345499 10.1017/S0007114517000204

[160] Ferrere G Wrzosek L Cailleux F Turpin W Puchois V Spatz M . Fecal microbiota manipulation prevents dysbiosis and alcohol-induced liver injury in mice. J Hepatol. 2017;66:806–15. 27890791 10.1016/j.jhep.2016.11.008

[161] Zhou D Pan Q Shen F Cao H Ding W Chen Y . Total fecal microbiota transplantation alleviates high-fat diet-induced steatohepatitis in mice via beneficial regulation of gut microbiota. Sci Rep. 2017;7:1529. 28484247 10.1038/s41598-017-01751-y PMC5431549

[162] Philips CA Pande A Shasthry SM Jamwal KD Khillan V Chandel SS . Healthy donor fecal microbiota transplantation in steroid-ineligible severe alcoholic hepatitis: a pilot study. Clin Gastroenterol Hepatol. 2017;15:600–2. 27816755 10.1016/j.cgh.2016.10.029

[163] Bajaj JS Kassam Z Fagan A Gavis EA Liu E Cox IJ . Fecal microbiota transplant from a rational stool donor improves hepatic encephalopathy: A randomized clinical trial. Hepatology. 2017;66:1727–38. 28586116 10.1002/hep.29306 PMC6102730

[164] Gerding DN Meyer T Lee C Cohen SH Murthy UK Poirier A . Administration of spores of nontoxigenic Clostridium difficile strain M3 for prevention of recurrent C. difficile infection: a randomized clinical trial. JAMA. 2015;313:1719–27. 25942722 10.1001/jama.2015.3725

[165] Dhillon TS Dhillon EK Chau HC Li WK Tsang AH . Studies on bacteriophage distribution: virulent and temperate bacteriophage content of mammalian feces. Appl Environ Microbiol. 1976;32:68–74. 987749 10.1128/aem.32.1.68-74.1976 PMC170007

[166] Duan Y Llorente C Lang S Brandl K Chu H Jiang L . Bacteriophage targeting of gut bacterium attenuates alcoholic liver disease. Nature. 2019;575:505–11. 31723265 10.1038/s41586-019-1742-x PMC6872939

[167] Scott AJ Alexander JL Merrifield CA Cunningham D Jobin C Brown R . International Cancer Microbiome Consortium consensus statement on the role of the human microbiome in carcinogenesis. Gut. 2019;68:1624. 31092590 10.1136/gutjnl-2019-318556 PMC6709773

[168] Butt J Jenab M Werner J Fedirko V Weiderpass E Dahm CC . Association of pre-diagnostic antibody responses to escherichia coli and bacteroides fragilis toxin proteins with colorectal cancer in a european cohort. Gut Microbes. 2021;13:1–14. 10.1080/19490976.2021.1903825 PMC8078709 33874856

[169] Vogl T Klompus S Leviatan S Kalka IN Weinberger A Wijmenga C . Population-wide diversity and stability of serum antibody epitope repertoires against human microbiota. Nat Med. 2021;27:1442–50. 34282338 10.1038/s41591-021-01409-3

[170] Sinha R Abu-Ali G Vogtmann E Fodor AA Ren B Amir A . Assessment of variation in microbial community amplicon sequencing by the microbiome quality control (MBQC) project consortium. Nat Biotechnol. 2017;35:1077–86. 28967885 10.1038/nbt.3981 PMC5839636

[171] Vandeputte D De Commer L Tito RY Kathagen G Sabino J Vermeire S . Temporal variability in quantitative human gut microbiome profiles and implications for clinical research. Nat Commun. 2021;12:6740. 34795283 10.1038/s41467-021-27098-7 PMC8602282

[172] Oehler N Volz T Bhadra OD Kah J Allweiss L Giersch K . Binding of hepatitis B virus to its cellular receptor alters the expression profile of genes of bile acid metabolism. Hepatology. 2014;60:1483–93. 24711282 10.1002/hep.27159

[173] Lopera-Maya EA Kurilshikov A van der Graaf A Hu S Andreu-Sánchez S Chen L . Effect of host genetics on the gut microbiome in 7,738 participants of the Dutch Microbiome Project. Nat Genet. 2022;54:143–51. 35115690 10.1038/s41588-021-00992-y

